# Rapid in-plate screening of biotransformation products in single zebrafish embryos†

**DOI:** 10.1039/d1ra01111a

**Published:** 2021-08-16

**Authors:** Anton Ribbenstedt, Jonathan P. Benskin

**Affiliations:** Department of Environmental Science, Stockholm University Sweden Anton.Ribbenstedt@aces.su.se

## Abstract

A procedure was developed for rapid screening of xenobiotic biotransformation products (bioTPs) in single zebrafish (ZF; *Danio rerio*) embryos. Exposure was carried out from 0–120 hours post fertilization (hpf) to 6 different concentrations of the model compound propranolol (PPL). Following in-plate extraction and non-target instrumental analysis by high resolution mass spectrometry, suspected bioTPs were identified using custom data filtration scripts and matching to *in silico* structural predictions. A total of eight PPL bioTPs were identified (five at a level 1 confidence and one at a level 2–3 confidence). These findings supplement previously generated toxicometabolomic models derived from the same dataset, and were obtained without conducting additional exposure experiments. In addition to facilitating assessments of inter-individual variability in bioTP production in ZF embryos, we demonstrate that bioTPs can be elucidated using extremely small quantities of biomass (*i.e.* ∼200 μg). To the best of our knowledge, this is the first time bioTP elucidation has been carried out in single ZF embryos.

## Abstract 2

A procedure was developed for rapid screening of xenobiotic biotransformation products (bioTPs) in single zebrafish (ZF; *Danio rerio*) embryos.

## Graphical abstract

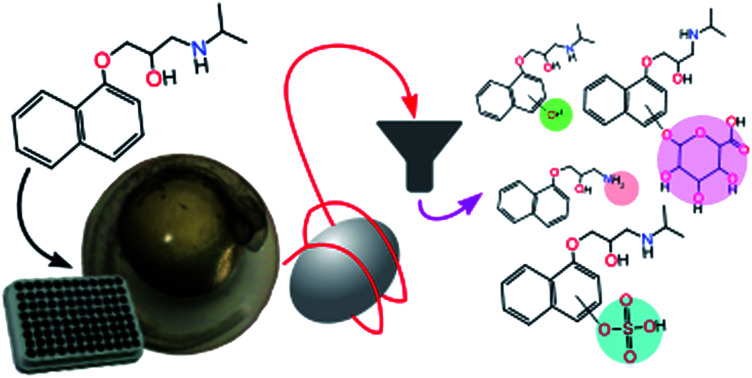

## 1 Introduction

Determination of pharmaceutical biotransformation is of great importance from both a pharmacotoxicological and environmental toxicological perspective. In pharmacotoxicology it is essential to establish that a drug does not generate toxic biotransformation products (bioTPs) within patients, to which end the European Medicinal Agency (EMA) and the US Food and Drug Administration (FDA) have both issued regulatory documents underlining the importance of detection and quantification of drug bioTPs during development of pharmaceuticals.^1,2^ Drug bioTPs may also form during wastewater treatment and are commonly detected in surface water downstream from waste-water treatment plants (WWTPs).^3^ In addition to the risks posed by bioTPs entering the aquatic environment, these substances have the potential to revert back to their parent compound either during wastewater treatment or by microbial activity in river sediment after release.^3^ Moreover, identification of bioTPs is important because some chemicals may only exert toxicity post-biotransformation.^4^ For example, biotransformation of acetaminophen can lead to formation of the hepatotoxic metabolite *N*-acetyl-*p*-benzoquinone imine.^5^

The non-selective beta-blocker propranolol (PPL) was first synthesized in the 1960s and is heavily prescribed to patients suffering from a range of heart-related diseases. The extensive use of PPL has led to its global occurrence in natural waters downstream from WWTPs.^6–9^ While as little as ∼16% of orally administered PPL is excreted unchanged, few studies have measured PPL bioTPs in the aquatic environment and only a single study has attempted identification of PPL bioTPs in fish.^10–17^ In that work, 5 bioTPs were identified, but structures remained tentative due to a lack of high resolution data or authentic standards. Further investigations into the formation and occurrence of PPL bioTPs are clearly important because PPL and mixtures of PPL bioTPs (produced through S9 incubation) have previously been shown to have similar biological effects.^10,18,19^

Due to their high genetic similarity to humans, zebrafish (ZF; *Danio rerio*) are frequently used to study human disease. Conservation of many common enzyme homologues between humans and ZF has led to an increased use of ZF of all life stages for studying xenobiotic biotransformation.^20–26^ Assays involving ZF embryos, which are considered an *in vitro* model under European Union legislation, are advantageous compared to their *in vivo* counterparts due to reductions in time, cost, and animal usage.^27^*In vitro* screening of bioTPs of aquatic pollutants have thus far predominantly been carried out using S9 liver fractions from fish.^28–30^ Although these tests share similar benefits to ZF embryos (relative to *in vivo* studies), they only consider biotransformation in the liver and do not account for uptake and elimination processes. To the best of our knowledge, all ZF embryo biotransformation experiments performed to date have involved pooled embryos and require a second experimental setup when combined with toxicological studies.^21–26,31–35^ While pooling of embryos can be advantageous for detecting lower abundance bioTPs, cost and throughput may be improved by using single embryos. Moreover, single embryo screening offers the opportunity to connect apical endpoints to extreme transformation profiles in individuals, and to measure inter-individual variability in biotransformation.

In this study we developed a rapid screening procedure for xenobiotic biotransformation in single ZF embryos using existing non-target data from a toxicometabolomic study involving PPL.^36^ By utilizing *in silico* bioTP structural predictions together with in-house R-scripts, we filtered out PPL bioTPs and confirmed their structures by matching to MS2 data (both *in silico* predictions and authentic standards). To the best of our knowledge, this is the first time bioTP elucidation has been carried out in single ZF embryos and highlights the potential for re-purposing non-target metabolomics data for bioTP identification and for extracting information on bioTPs from extremely small sample sizes.

## 2 Materials and methods

### 2.1 Standards

PPL (99% purity), *N*-desisopropyl propranolol (>97.5% purity; DIP-PPL) and 4-hydroxypropranolol hydrochloride (98.5% purity; PPL-4OH) were purchased from Merck (Darmstadt, Germany). 4-Hydroxypropranolol sulfate potassium (98% purity; PPL-OH-SO_4_), propranolol glucuronide (96.2%; PPL-glucu), 4-hydroxypropranolol glucuronide (99.2%; PPL-OH-glucu) was purchased from ALSACHIM (Shimadzu, Kyoto, Japan). 1-Naphthol (also known as 1-naphthoic acid; 98% purity) was purchased from Alfa Aesar (Thermo, USA). All other standards and reagents used in this study are described in detail elsewhere.^36^

### 2.2 Zebrafish embryo incubation and extraction

Embryo exposures have been described in detail in our prior publication; additional exposures were not carried out for the present work.^36^ Briefly, single ZF embryos were exposed to six concentrations of PPL (0.050, 0.49, 12, 62, 4550 and 46 540 μg L^−1^; *n* = 12 embryos per dose), 40 000 μg L^−1^ of the positive control 3,4-dichloroaniline (12 embryos) and to clean tank water (12 embryos) in a 96-well plate (wp) from 0–120 hours post fertilization (hpf). At termination, exposure water was removed and embryos were subsequently frozen on dry ice, transported to Stockholm University and stored at −80 °C. Extraction was carried out by homogenizing the embryos in-plate using mixed-size, stainless steel beads together with a mixture of methanol and chloroform containing a metabolite internal standard (Sphingomyelin ([d18:1/12:0]; see ref. 36 for details)). Post-homogenization the plates were sonicated and centrifuged prior to placing them directly into the auto-sampler for instrumental analysis. Further details on sample extraction are provided elsewhere.^36^

### 2.3 Instrumental analysis

Details of the instrumental analysis have been described previously.^36^ A brief overview is provided here: the concentration of PPL in each dose was determined by liquid chromatography-tandem mass spectrometry (LC-MS/MS) by injecting exposure water collected from individual wells of the 96-well plate.^36^ Non-target data, which were re-interrogated in the present work for bioTPs, were obtained by injecting embryo extracts onto an Ultimate 3000 LC equipped with an in-line filter prior to the chromatographic column. The LC was connected to a QExactive HF Orbitrap high resolution mass spectrometer (HRMS; Thermo, USA) operated in full scan with data-dependent MS2 acquisition, based on the three most intense peaks per full scan. Two analyses were performed per sample, utilizing either a BEH amide hydrophilic interaction liquid chromatography (HILIC) column (Waters, USA) with the MS operated in positive electrospray ionization (ESI) mode, or a T3 Atlantis C18 column (Waters) with the MS operated in negative ESI mode.^36,37^

### 2.4 Data analysis

Compound Discoverer 3.1 (CD; ThermoFisher Scientific, USA) was used for non-target peak picking and retention time (RT) alignment. Peak picking was configured to only approve compounds with one matching isotopic peak and to combine peaks determined to be adducts into a single feature. The workflow employed for processing in CD also included the “*Generate expected compounds*”-node which predicts exact masses of potential phase I and II bioTPs of a structure supplied by the user (see Text S1 for details†). This processing step also annotates bioTP predictions. Thereafter, feature areas were exported to the statistical software R wherein replicates displaying lethal and severe morphological endpoints were filtered out.^38^ We then removed features that produced a signal in more than 1 sample for which no PPL was added (*i.e.* the positive and negative control samples; *n* = 24). The limit of 1 was set in order to avoid removing real PPL bioTPs due to noise present in a single sample. Finally, we calculated an enrichment factor (EF) by dividing each feature's normalized area in the highest dose (where bioTPs are expected to occur in highest abundance) by its average area in negative controls. Features with EFs ≤10 were subsequently removed. All remaining features were then manually scrutinized in CD to confirm that features of interest were absent in control samples, that the signal in the exposed samples had a logical gradient (*i.e.* gradual increase in abundance of the bioTP with dose), and to assess MS2 data. If a theoretical bioTP mass (as predicted by CD) had several possible transformation pathways, the one with the highest fragment ion searching (FISh)-score (*i.e.* how well fragments match to an *in silico* generated collision-induced dissociation (CID) pattern of the suggested chemical structure) was selected. Finally, to obtain a less biased structural confirmation of the identities proposed by CD, MS2 data from the highest two doses were converted into mzXML-format prior to analysis using SIRIUS + CSI:FingerID (referred to herein as simply “Sirius”).^39–41^ Sirius utilizes MS1 and MS2 spectra to predict chemical formulae for parent compounds and their fragments and compares the predictions to a range of databases (see Table S1† for settings used). The output is a list of chemicals which fit the MS2 spectrum of interest and a description of how well substructures of the molecule fit substructures of *in silico* fragmentation spectra of the chemicals in that list. Sirius is a powerful software for predicting bioTP structure of chemicals but is computationally demanding and time consuming when using the Java interface on large, unfiltered datasets. Although automatic annotation software exists (such as the R-package patRoon), it requires the user to optimize peak picking parameters prior to usage.^42^ Since the data in our study was processed using CD, the time investment to fully optimize XCMS or OpenMS settings would be comparable to simply using Sirius. However, when combined with the bioTP prediction capability of CD and the prioritization workflow outlined above, the time required to utilize Sirius for structure predictions is markedly decreased. Lastly, all identified bioTPs were re-processed using XCalibur 3.0.63 (Thermo, USA) since this software was more effective than CD for manual inspection of MS2 data, for assessing the occurrence of in-source fragments and peak integration. All correlation analyses were carried out using Spearman's rank order correlation tests.

### 2.5 BioTP structural confirmation

Structures identified using CD and Sirius were confirmed by analyzing authentic standards (when available) fortified into both pure solvent and also non-exposed ZF embryo extracts (to assess the effect of matrix on retention times). These standards were then analyzed under the same instrumental conditions that were used for analysis of the original embryo extracts.^36^

The R-script MSMSsim was used to compare MS2 data obtained from standards to that of samples (see Table S2† for settings).^43^ MSMSsim assesses the similarity of two spectra, within a time window specified by the user, and provides a score between 0 (no similarity) and 1 (identical). Scores ≥90% were considered sufficient to conclude a positive match between standard and sample. The scale described by Schymanski *et al.* was used to denote the level of confidence (CL) in the molecular structures.^44^

## 3 Results and discussion

### 3.1 BioTP prioritization

The number of raw features obtained from CD amounted to 7370 and 6173 from the +ve HILIC and −ve C18 LC-HRMS analyses, respectively. From these features, a total of 5705 neutral masses were identified as potential bioTPs by CD, highlighting the need for a prioritization strategy. For the HILIC dataset, application of the ExpMet script reduced the number of features to 727 plausible bioTPs, which was further reduced to 627 after filter 1 and to 14 after filter 2. For the C18 dataset the same procedures led to 738 features after ExpMet, 548 after filter 1 and 6 features after filter 2. Overall, a combined total of 20 plausible bioTPs (consisting of 13 exact masses) were obtained from both datasets. The neutral masses (Da) for these suspects were: 133.11014 (2 features in HILIC), 178.08328 (C18), 182.07288 (HILIC), 217.1102 (2 features in HILIC [average mass from both features]), 274.15269 (HILIC), 275.15218 (2 features in HILIC), 293.16258 (HILIC), 355.10882 (HILIC + C18), 396.17211 (HILIC), 435.18913 (HILIC + C18), 451.18410 (HILIC + 2 features in C18), 635.27319 (C18) and 651.26804 (HILIC).

### 3.2 BioTP identification

Among the 20 features obtained following prioritization, the mass at 178.08328 Da (C18) was removed due to its prevalence in blanks. The remaining features were subjected to further investigation using Sirius together with FISh scoring and matching in mzCloud (when possible). For one of the features at 217.11029 Da observed in HILIC (RT = 3.431 min), the PPL bioTP DIP-PPL was the top prediction in 100% of MS2 spectra investigated by Sirius. FISh scoring revealed matches to DIP-PPL for 8 fragments (50% of total MS2 fragments; see Fig. S1†) for this feature. Acquisition of an authentic standard confirmed that it was indeed DIP-PPL (>99% match by MSMSsim; CL = 1; see Fig. S2†). The second feature at 217.11029 Da (RT = 3.307 min) did not generate any MS2 spectra and was assigned a CL of 5.

For the mass 275.15218 Da, two features were observed in HILIC (RT = 3.194 and 3.269 min). For the feature at 3.194 min, PPL-4OH was the top prediction in 100% of investigated MS2 spectra by Sirius. PPL-4OH was also matched by mzCloud (94.6%) and 18 fragments (100%; see Fig. S3†) in the MS2 spectrum were annotated by FISh scoring as fragments of PPL-4OH. The feature at 3.269 min was, however, also predicted to be PPL-4OH in 100% of the samples by Sirius and 20 fragments (61%; see Fig. S4†) were annotated by FISh scoring. Following acquisition of an authentic standard of PPL-4OH, we confirmed the presence of this PPL bioTP at 3.194 min (>96% match by MSMSsim; CL = 1; see Fig. S5†). The feature at 3.269 min is assumed to be PPL-5OH (CL = 3–4), due to the very similar RT, exact mass and the high similarity between the MS2 spectra when compared using MSMSsim (see Fig. S6†).

**Fig. 1:**
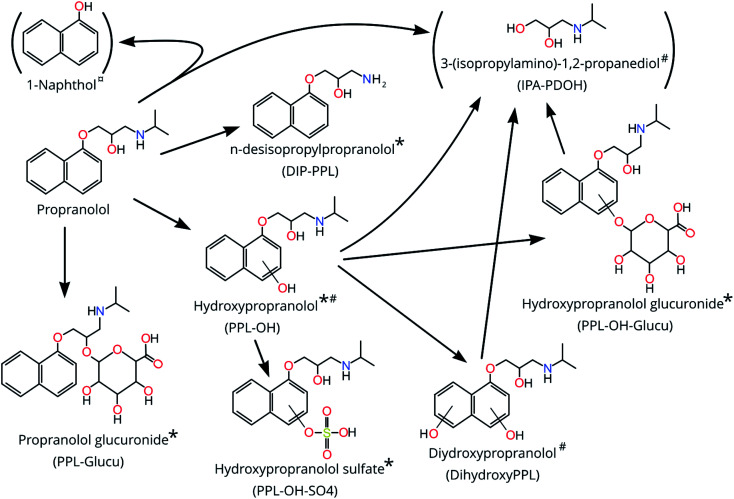
The biotransformation products of propranolol detected and determined with level 1–3 confidence level (CL) after exposure of zebrafish embryos to propranolol for 120 hpf and their metabolism pathways from propranolol. * = CL 1; # = CL 3–4; ¤ = CL 5. Structures in parenthesis could not be confirmed as occurring exclusively from biotransformation of PPL and are therefore considered tentative.

The neutral mass 355.10882 was observed in both HILIC (*m*/*z* 356.11588) and C18 (*m*/*z* 354.10165). Both features were strongly correlated (*r*_s_ = 0.97) suggesting that they belong to the same substance. Sirius revealed that the top match for all MS2 spectra (100% for both HILIC and C18) was the bioTP PPL-OH-SO_4_ (see Fig. 1). However, only MS2 data acquired by HILIC resulted in FISh annotation (24 fragments [75% of total] ascribed to PPL-OH-SO_4_; see Fig. S7†). Using an authentic standard and MSMSsim we were able to confirm a structural match for PPL-OH-SO_4_ in both C18 (>96%; CL = 1; see Fig S8†) and HILIC (>99%; CL = 1; see Fig S9†). While formation of PPL-OH-SO_4_ is well documented in humans and other mammals, to the best of our knowledge this is the first study to observe this bioTP in fish.^45–47^

For the features at 133.110 Da (HILIC; RT = 3.668 and 3.828 min) the identity suggested by CD was 3-(isopropylamino)-1,2-propanediol (IPA-PDOH; See Fig. 1) and 8 fragments for both features were matched through FISh scoring (80% for both; see Fig. S10 and S11†). The top Sirius prediction for all of the MS2 spectra was 2-(propan-2-ylamino)propane-1,3-diol, which has the same chemical formula as IPA-PDOH. Furthermore, IPA-PDOH was among the top 5 Sirius predictions in 100% of the MS2 spectra for both of the features occurring at 3.668 and 3.868 min. Close inspection of chromatograms revealed that RTs were dissimilar to PPL and other bioTPs, which ruled out in-source fragmentation. However, the exposure medium contained a feature which matched the feature at 3.868 min, so we could not unequivocally confirm IPA-PDOH as a bioTP. Considering the matches by FISh-scoring and Sirius, together with the knowledge that IPA-PDOH is a plausible bioTP, we ascribed a CL of 3–4 for the feature at 3.868 min. Identification of IPA-PDOH prompted a search for its expected co-bioTP 1-naphthol, the exact mass of which (144.05733) did not appear among the final 11 suspects selected from our prioritization approach. Upon further inspection, an exact mass consistent with 1-naphthol appears to have been removed during prioritization due to its low EF. Analysis of an authentic standard did not provide confirmation of the structure due to an interference (present in the samples) with similar nominal mass which produced overlapping MS2 spectra that could not be deconvoluted.

For the neutral mass at 293.163 Da (HILIC), Sirius suggested the PPL bioTP dihydroxyPPL as the 4th highest ranked suggestion for this feature. Moreover, FISh scoring successfully matched all 12 fragments (100%; Fig. S12†) in the experimentally-derived MS2 spectrum to dihydroxyPPL. Consequently, this structure was assigned a CL of 3–4.

The neutral masses at 435.189 Da (HILIC + C18) and 451.184 Da (HILIC + 2 features in C18) were suggested by CD to be glucuronidated phase 2 conjugates. For a given neutral mass, peak intensities obtained from C18 and HILIC were significantly and highly correlated (*r*_s_ >0.83; *p* <0.005) suggesting that the same substance was observed in both analyses, and possibly the presence of isomers in the case of the 2 features at 451.184 in C18. Sirius results further supported the hypothesis of conjugation, with 100% of submitted MS2 spectra for the mass at 451.184 matching hydroxypropranolol glucuronide (PPL-OH-glucu) (all three features in HILIC and C18) and 100% of submitted MS2 spectra for 435.189 (HILIC and C18) matching propranolol glucuronide (PPL-glucu). For the features at 435.189, a total of 23 fragments in HILIC (57%; see Fig. S13†) and 3 fragments from C18 (27%; see Fig. S14†) were successfully matched to PPL-glucu using FISh scoring. Likewise, for the feature at 451.184, a total of 15 fragments were matched to PPL-OH-glucu in HILIC (53%; see Fig. S15†) while 8 and 6 fragments were matched to PPL-OH-glucu in C18 (32%; see Fig. S16;† 46%; see Fig. S17†) (for features at RTs 1.1 and 3.591 min, respectively). Using MSMSsim and authentic standards we were able to confirm both compounds, in C18 and HILIC at CL 1 (all 99%; see Fig. S18–S21†), with the possibility of an isomer explaining the 2nd feature for 451.184 Da in the C18 analysis.

The remaining 5 suspects (neutral masses 182.0729, 274.153, 396.172, 635.273 and 651.268 Da) could not be confirmed as PPL bioTPs. MS2 spectra were not collected for the feature at 274.153, while for 396.172 Da, matches in Sirius were inconsistent and did not include PPL bioTPs amongst the plausible structural predictions. For 182.0729 the Sirius predictions did include a plausible in-source fragment of PPL, and the retention times of PPL and this feature overlapped. For 651.273 the CD- and Sirius-predicted formulas agreed, and Sirius predicted all 11 MS2 spectra as a complex molecular structure without any obvious connection to PPL (see Table S3†). For 635.273 there was a mismatch between the prediction of chemical formula and only 67% of the 6 MS2 spectra agreed on a non-bioTP related complex molecular structure as the top prediction in Sirius.

### 3.3 Interindividual variability in bioTP production

**Fig. 2:**
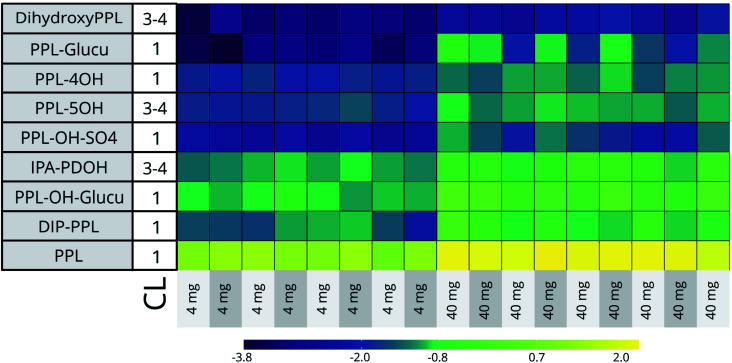
Heatmap showing log 10 transformed inter-individual variability in bioTP formation in the 4600 μg L^−1^ and 45 500 μg L^−1^ dose groups. CL = Confidence level. Acronyms: IPA-PDOH (3-isopropylamino-1,2-propanediol), DIP-PPL (*n*-desisopropylpropranolol), PPL-4 and 5OH (hydroxypropranolol), PPL-glucu (propranolol glucuronide), dihydroxyPPL (dihydroxypropranolol), PPL-OH-SO_4_ (hydroxypropranolol sulfate), PPL-OH-glucu (hydroxypropranolol glucuronide) and PPL (propranolol).

At the two highest doses (45 500 μg L^−1^ [*n* = 9] and 4600 μg L^−1^ [*n* = 8]) the RSDs of the 5 CL 1 identified bioTPs ranged from 21% (PPL-OH) to 109% (PPL-glucu in C18) across both HILIC and C18 datasets. Biotransformation capacity is known to vary significantly in humans, and has also been shown to vary in fish.^48–50^ Although the variation between individuals for some bioTPs in our data is considerable, the general profile of these compounds is comparable between embryos (See Fig. 2). Seeing how the conditions for the embryos are close to identical through the entire exposure, genetic polymorphism in individuals seems the most likely reason for the disparities in biotransformation efficiency.^48,49^

### 3.4 Implications for pharmacotoxicology and environmental toxicology

In this study a total of 7 confirmed PPL bioTPs were observed (5 at CL 1 and 2 at CL 3–4; see Table 1). Among these bioTPs, DIP-PPL, PPL-OH and PPL-OH-glucu have been previously observed in humans,^10^ mammals^46,47,51^ and fish.^17^ PPL-OH-SO_4_, PPL-glucu, dihydroxyPPL and multiple isomers of PPL-OH are here reported for the first time in fish. Two additional structures were also confirmed at CL2-3 and 4 (IPA-PDOH and 1-naphthol, respectively) but due to the observation of the former in the dosing medium and the latter as a (potential) in-source fragment of PPL, we could not confirm these structures as bioTPs of PPL. Overall, this is the first time bioTPs have been measured in single ZF embryos, and shows the potential for bioTP screening in very small samples. Elucidation of bioTP formation can contribute to the weight of ecotoxicological findings. Moreover, the potential to screen for bioTPs and metabolomic response in the same dataset from the same individual, increases throughout, reduces costs, and may facilitate links between bioTPs and toxicometabolomic perturbations in the same individual.

**Table 1:** Propranolol biotransformation products identified in the present work, including retention time (RT), mass error (dPPM), similarity score determined by MSMSsim, occurence in datasets, and confidence level (CL) in identification. Structures in parenthesis could not be confirmed as occurring exclusively from biotransformation of PPL

BioTPs	Exact mass	RT	dPPM	Similarity score	Datasets	CL	Comment
5OH-PPL	275.15231	3.269	0.33		HILIC	3–4	—
4OH-PPL	275.15205	3.194	0.61	0.996	HILIC	1	—
PPL-OH-SO_4_	355.109^a^	3.4	0.45	0.969	HILIC & C18	1	—
PPL-glucu	435.189^a^	3.7	0.41	0.993	HILIC & C18	1	—
PPL-OH-glucu	451.184^a^	4.0	0.35	0.953	HILIC & C18	1	Possible isomer
DIP-PPL	217.110	3.4	0.46	0.997	HILIC	1	—
DihydroxyPPL	293.163	3.6	0.44		HILIC	3–4	—
(IPA-PDOH)	133.110^a^	3.8	1.05		HILIC	3–4	Possible dose impurity
(1-Naphthol)	144.057	3.2	1.3		HILIC	5	—

a average of neutral masses measured in HILIC and C18.

## Data availability

All data used for this study are readily available through the Dryad Digital Repository (https://doi.org/10.5061/dryad.7m0cfxppz) and through contact with the corresponding author.

## Code availability

Code used for this study is available through GitHub (https://github.com/parasitetwin/ExpMetFilter) or through direct contact with the corresponding author.

## Author contributions

Anton Ribbenstedt: conceptualization, data curation, formal analysis, investigation, methodology, software, visualization, writing – original draft, writing – review & editing, Jonathan P. Benskin: conceptualization, formal analysis, project administration, supervision, validation, visualization, writing – original draft.

## Conflicts of interest

There are no conflicts to declare.

## Supplementary Material

RA-011-D1RA01111A-s001

RA-011-D1RA01111A-s002
