# Supplementary material for: Rapid in-plate screening of biotransformation products in single zebrafish embryos
Source: RSC Adv. 2021 Aug 16;11(45):27812–9. doi: 10.1039/d1ra01111a (PMC9038038; doi:10.1039/d1ra01111a)
Supplement: RA-011-D1RA01111A-s001 [file RA-011-D1RA01111A-s001.pdf]

Electronic Supplementary Material (ESI) for RSC Advances.
This journal is © The Royal Society of Chemistry 2021

Supplementary Information

Rapid in-plate screening of biotransformation products in single zebrafish embryos

Anton Ribbenstedt* and Jonathan P. Benskin

Department of Environmental Science and Analytical Chemistry (ACES), Stockholm University, Sweden

*Corresponding author:

Anton.Ribbenstedt@aces.su.se

Abbreviations used in this document in order of appearance:

Desisopropylpropranolol = DIP-PPL
4/5-hydroxypropranolol = 4/5-OH PPL
4"-hydroxypropranolol sulfate = PPL-OH-SO4
3-(isopropylamino)-1,2-propanediol = IPA-PDOH
Dihydroxypropranolol = DihydroxyPPL
Propranolol glucuronide = PPL-Glucu
Hydroxypropranolol glucuronide = PPL-OH-Glucu

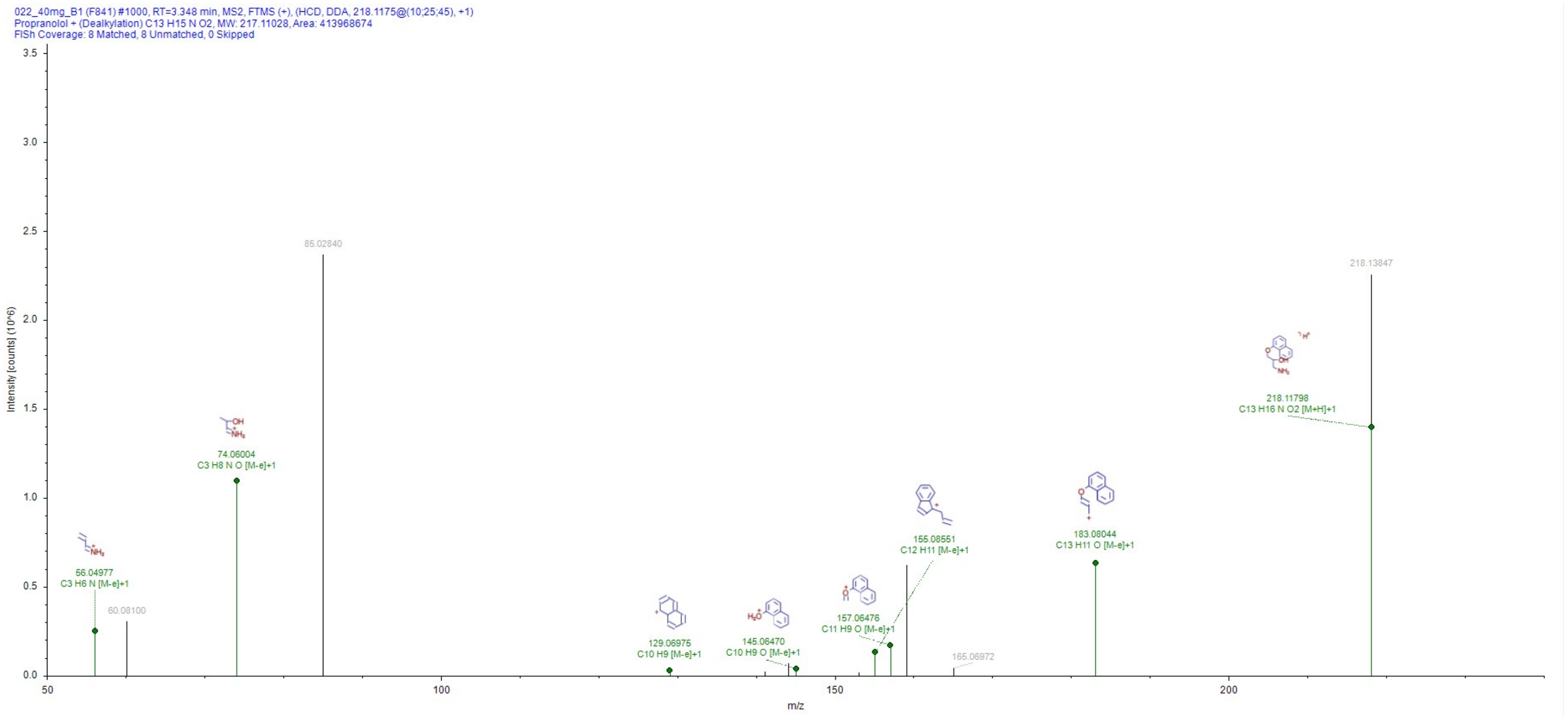

Figure S1. FISh scoring of feature 217.11040 (DIP-PPL), peak RT 3.431 from Compound Discoverer.

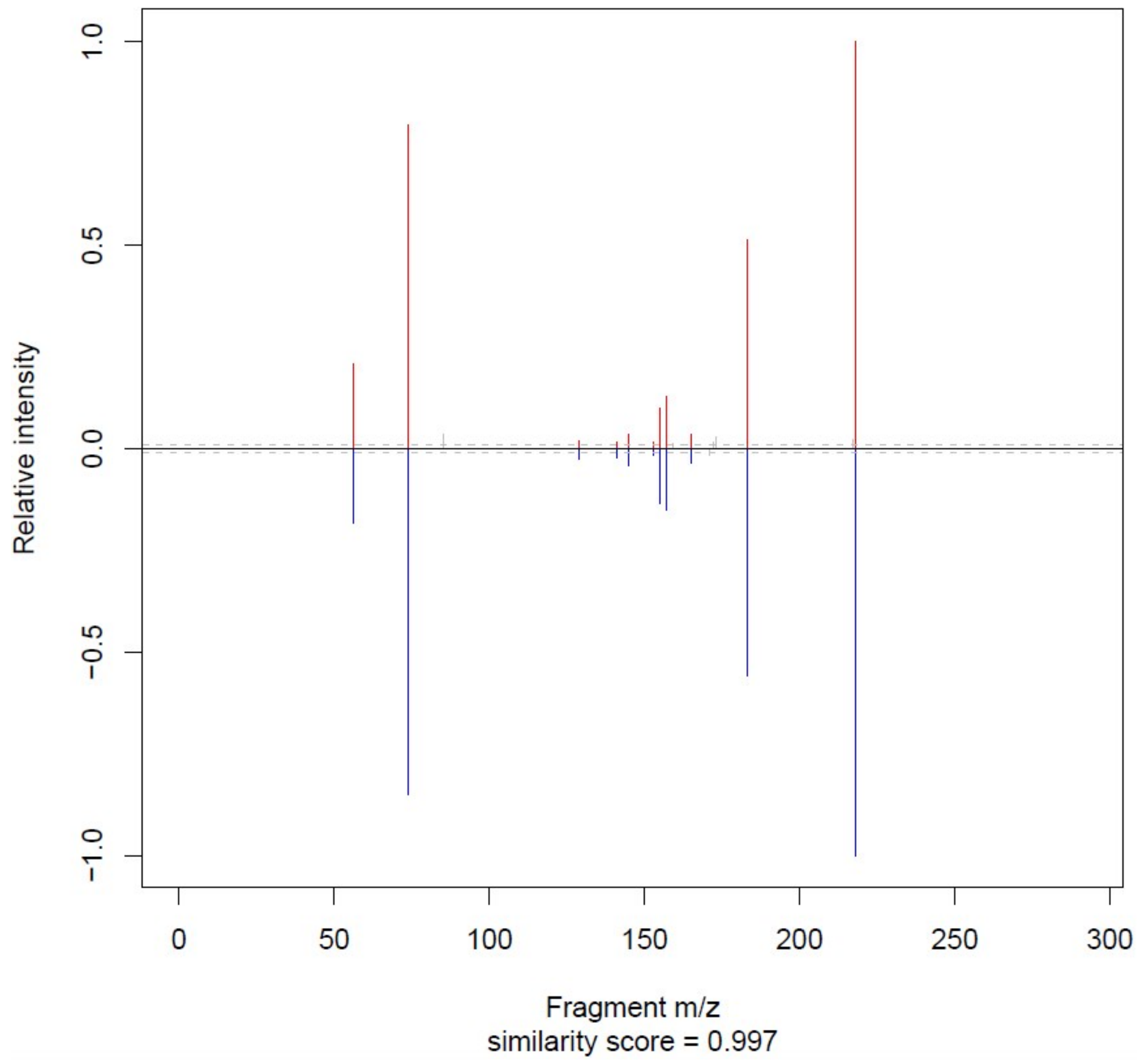

Figure S2. Similarity score from MSMSsim analysis of MS2 spectra of DIP-PPL from a sample (upper half) and an authentic standard (lower half).

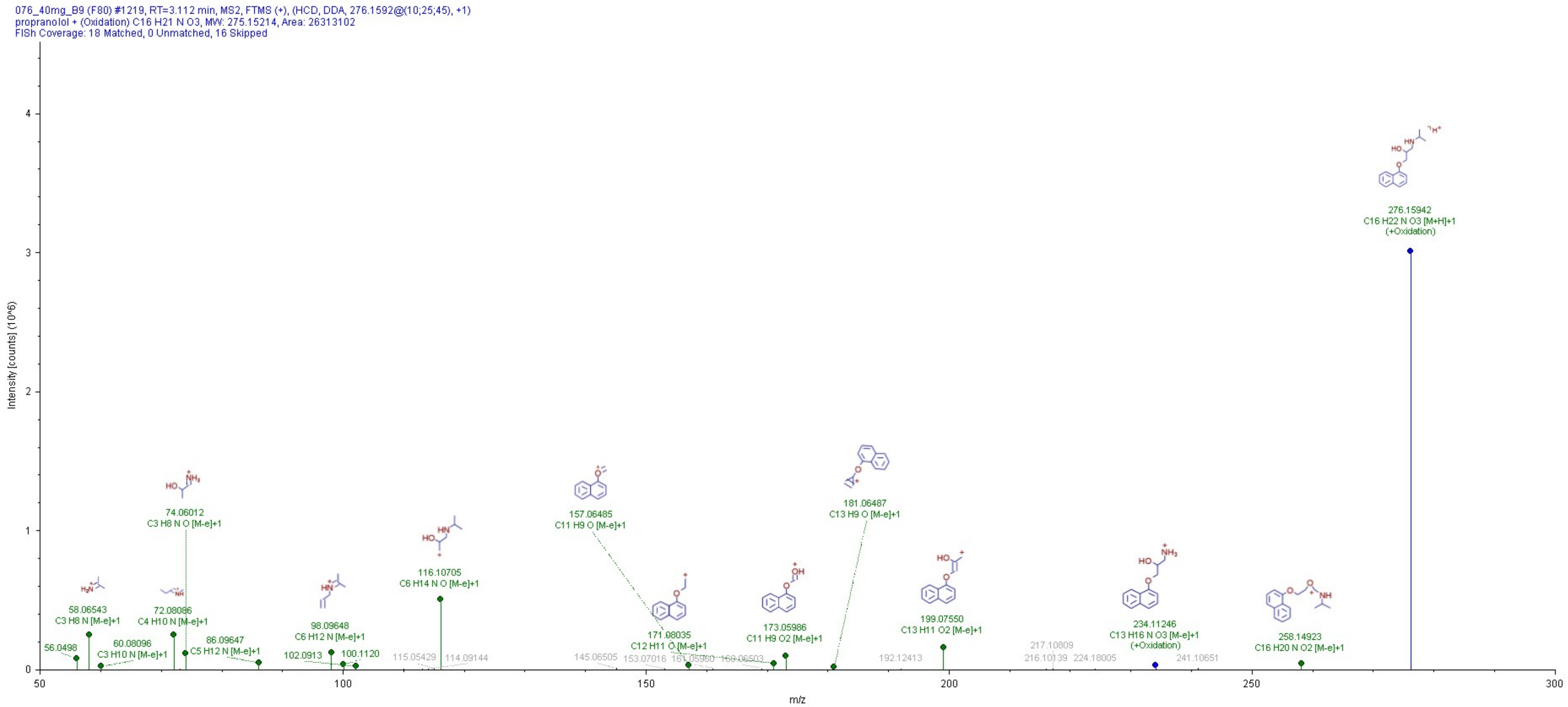

Figure S3. FISh scoring of feature 275.15231 (4-OH PPL), peak RT 3.194 from Compound Discoverer.

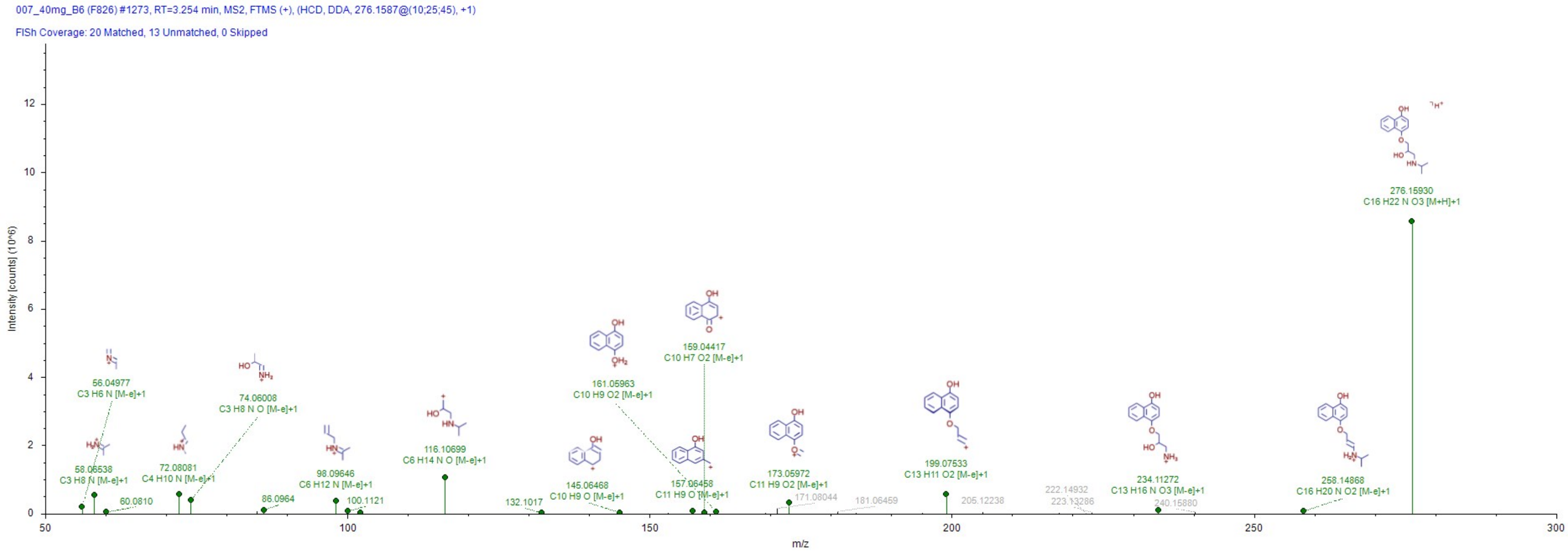

Figure S4. FISh scoring of feature 275.15231 (4-OH PPL), peak RT 3.269 from Compound Discoverer.

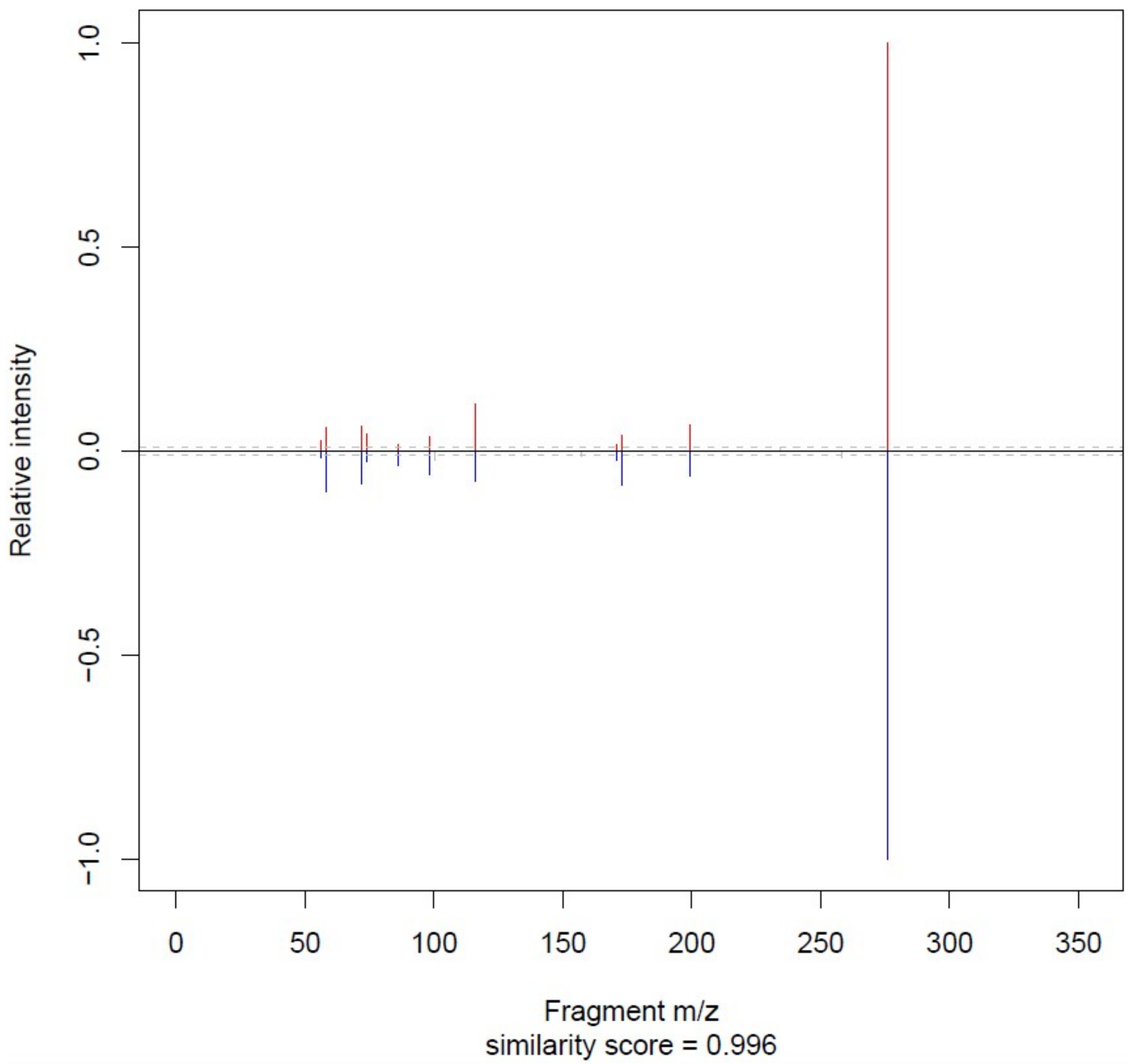

Figure S5. Similarity score from MSMSsim analysis of MS2 spectra of PPL-4OH at 3.194 from a sample (upper half) and an authentic standard (lower half).

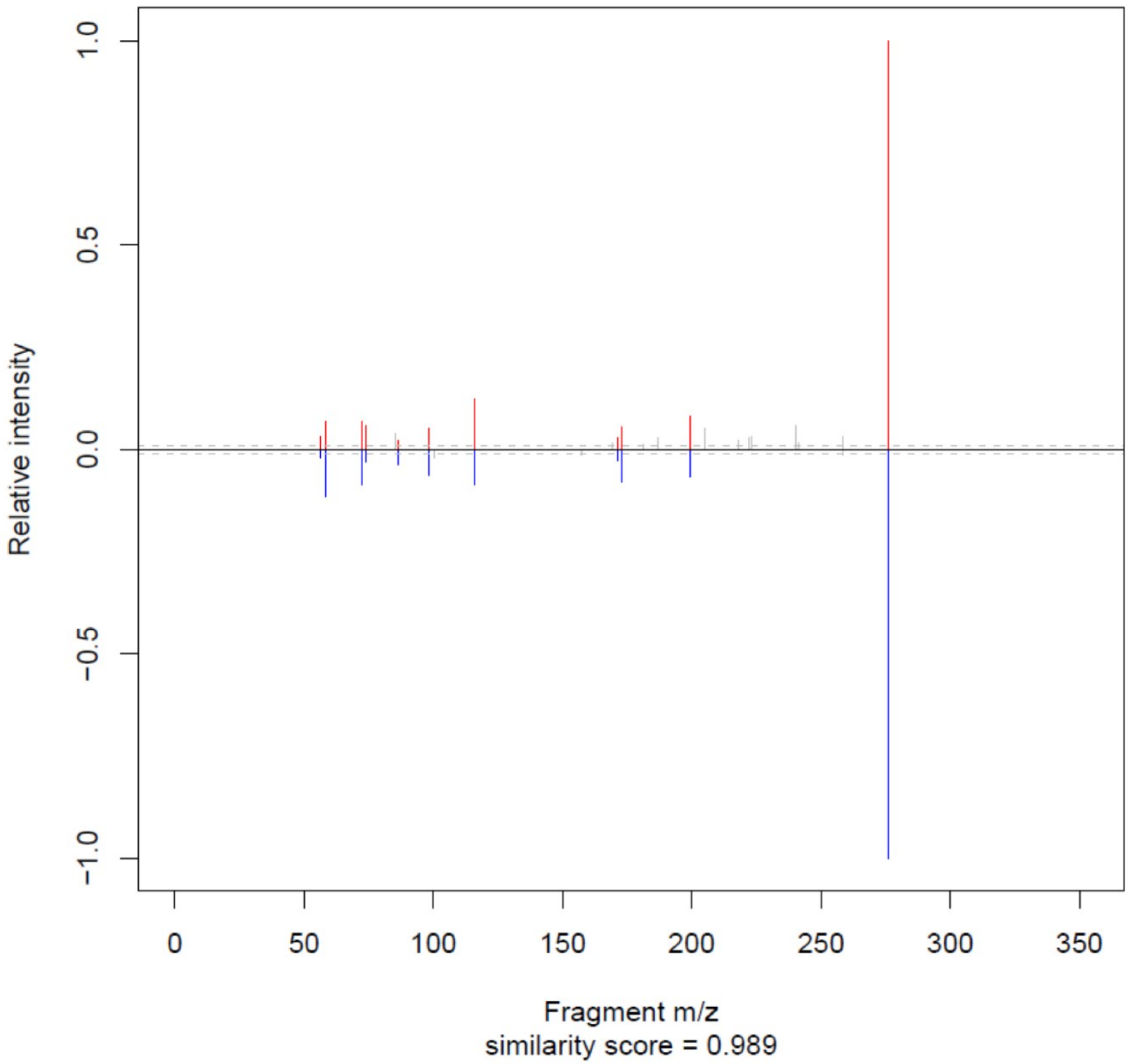

Figure S6. Similarity plot from MSMSsim analysis of MS2 spectra of 275.15218 Da at RT 3.269 min, believed to be PPL-5OH, from a sample (upper half) and an authentic standard of PPL-4OH (lower half). Portraying the high similarity as well as the subtle differences between the spectra.

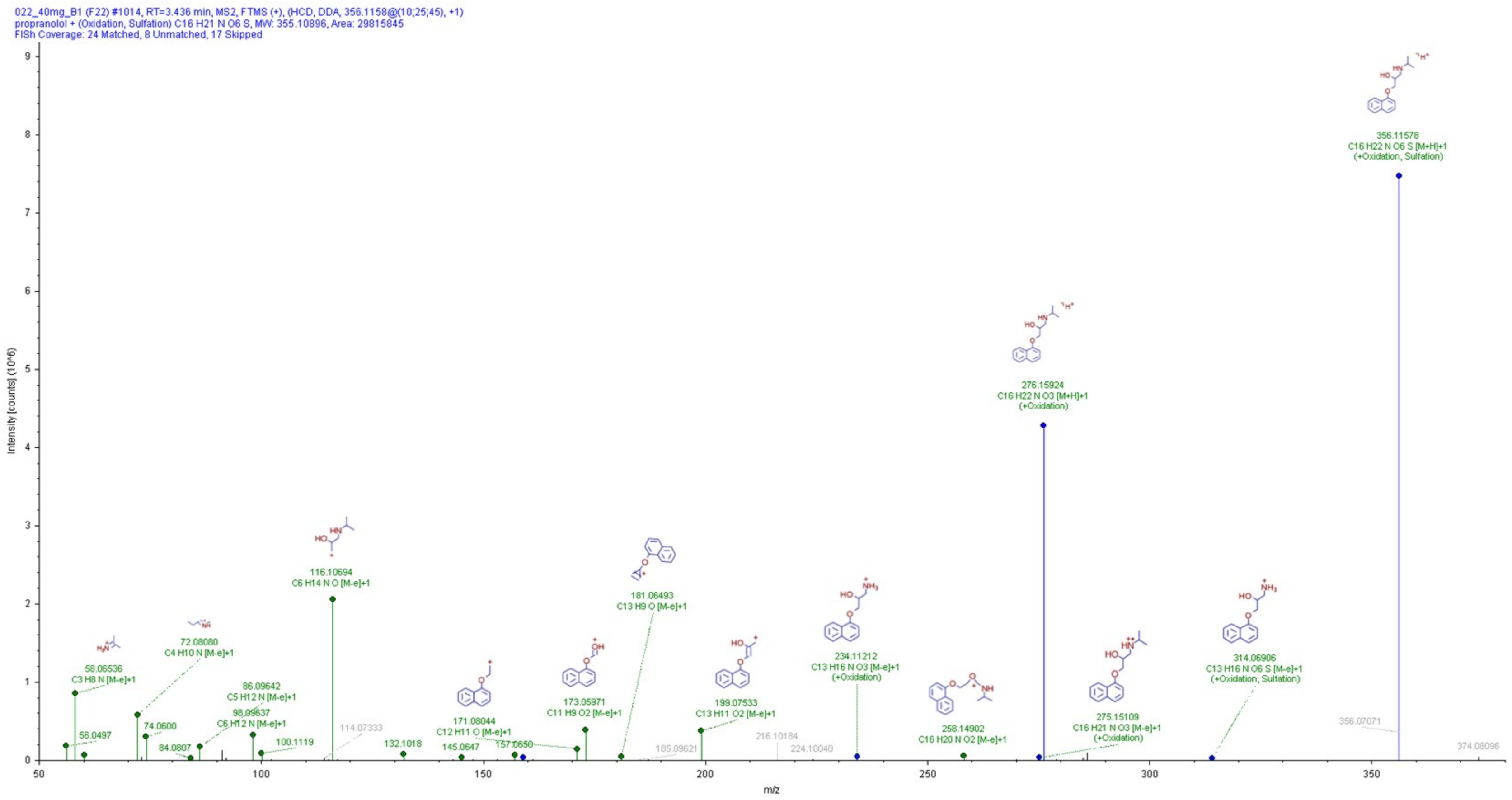

Figure S7. FISh scoring of feature 355.10880 (PPL-OH-SO4 ; HILIC) from Compound Discoverer.

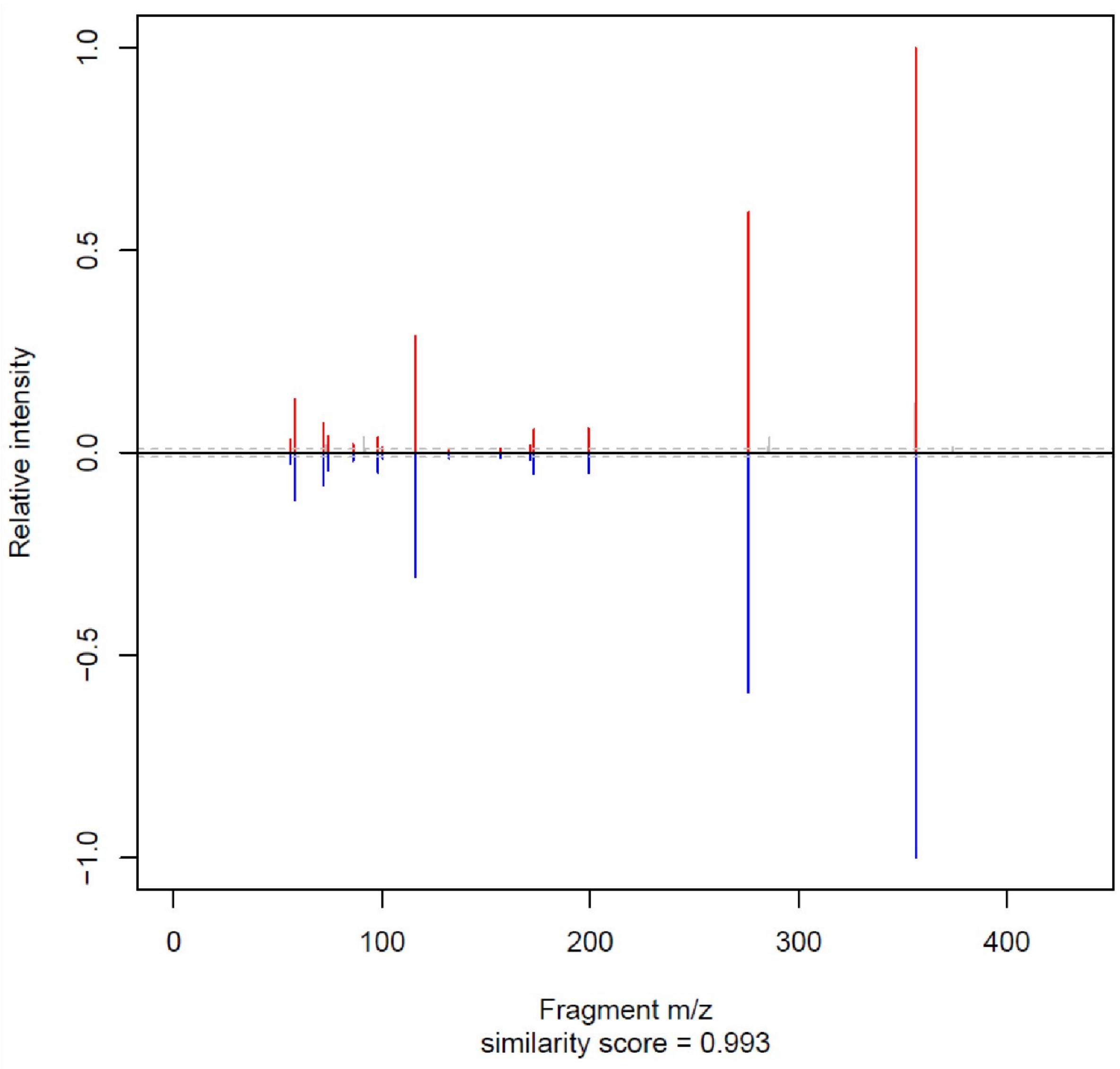

Figure S8. Similarity score from MSMSsim analysis of MS2 spectra of PPL-OH-SO4 (HILIC) from a sample (upper half) and an authentic standard (lower half).

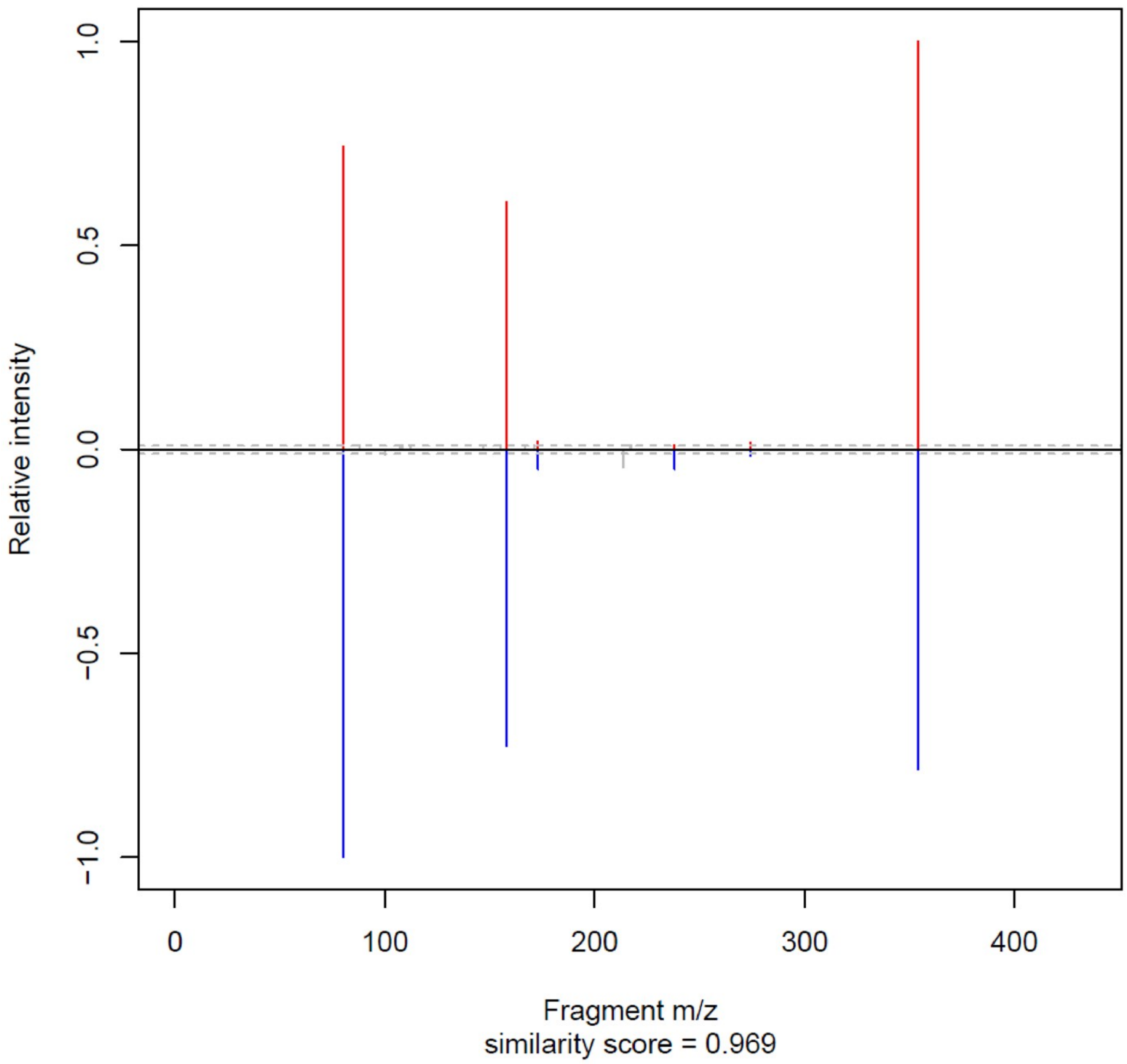

Figure S9. Similarity score from MSMSsim analysis of MS2 spectra of PPL-OH-SO4 (C18) from a sample (upper half) and an authentic standard (lower half).

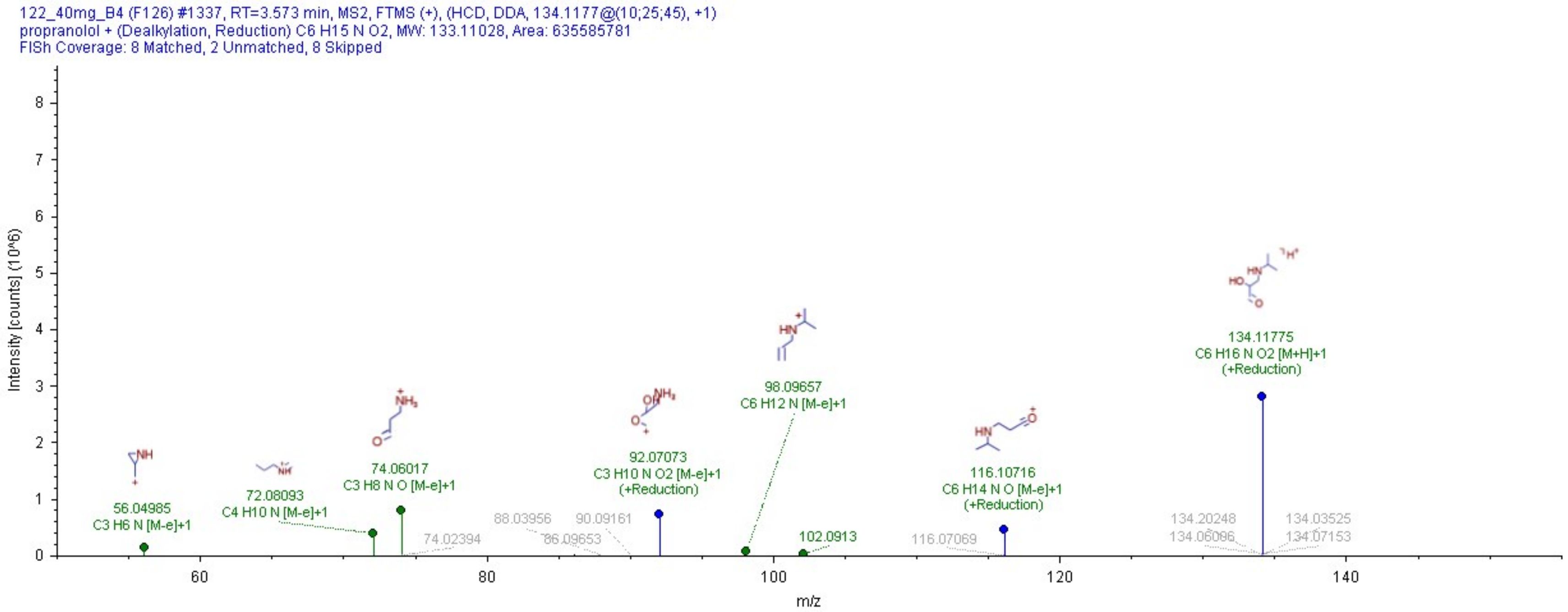

Figure S10. FISh scoring of feature 133.16258 (IPA-PDOH) at RT 3.828 min from Compound Discoverer.

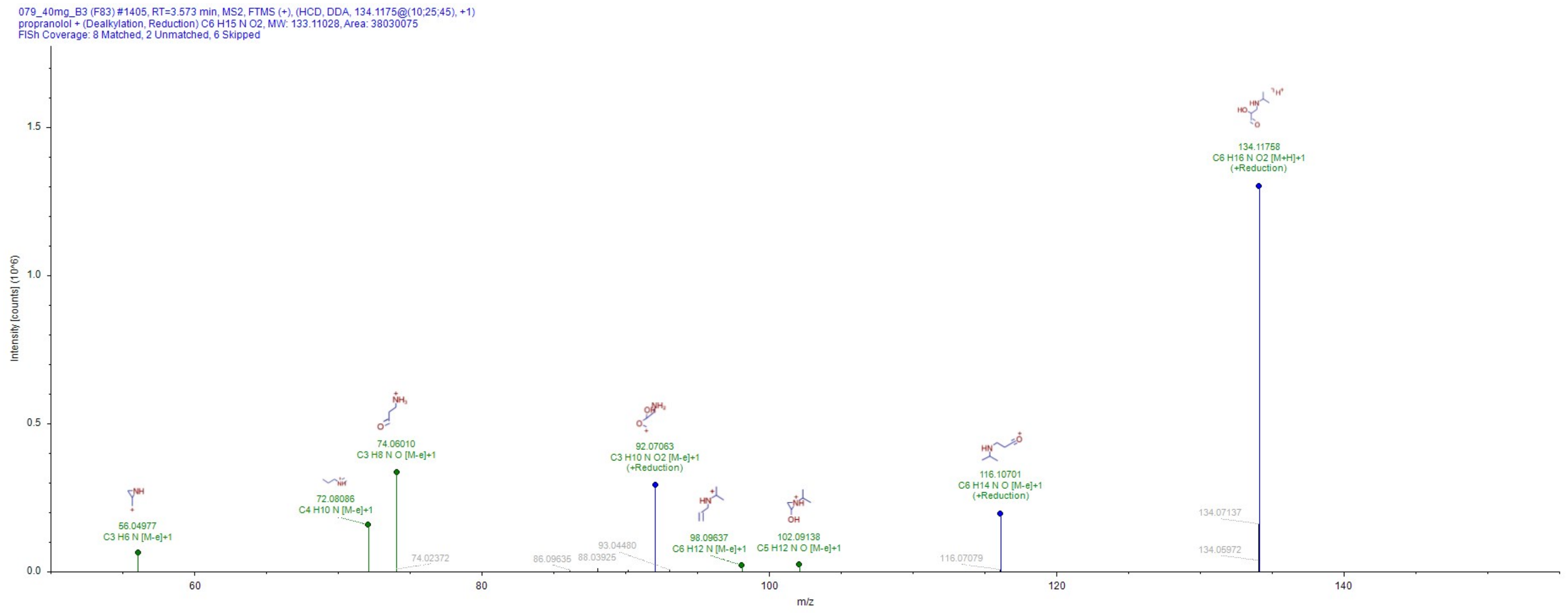

Figure S11. FISh scoring of feature 133.16258 (IPA-PDOH) at RT 3.668 min from Compound Discoverer.

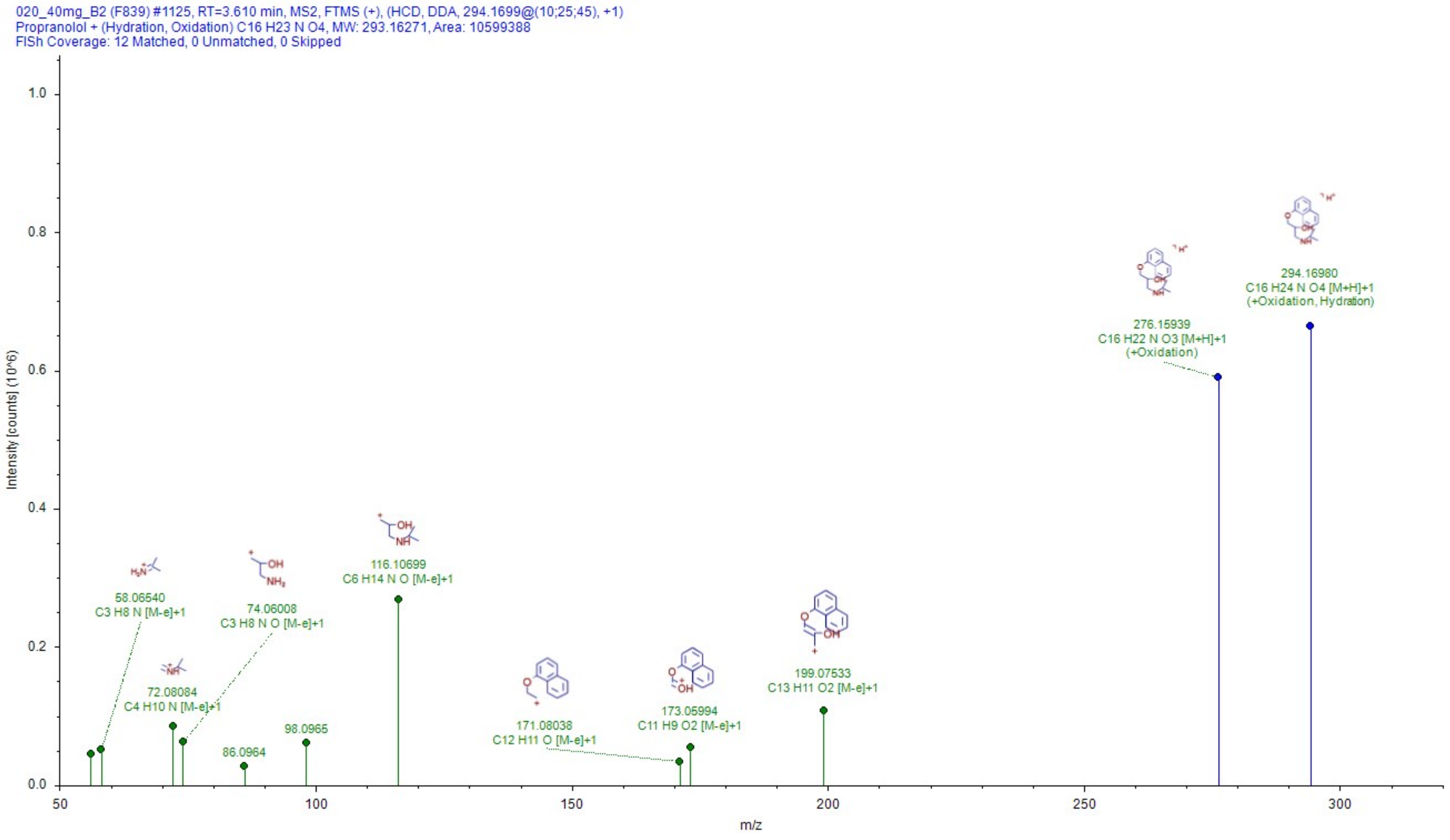

Figure S12. FISh scoring of feature 293.16258 (DihydroxyPPL; HILIC) from Compound Discoverer.

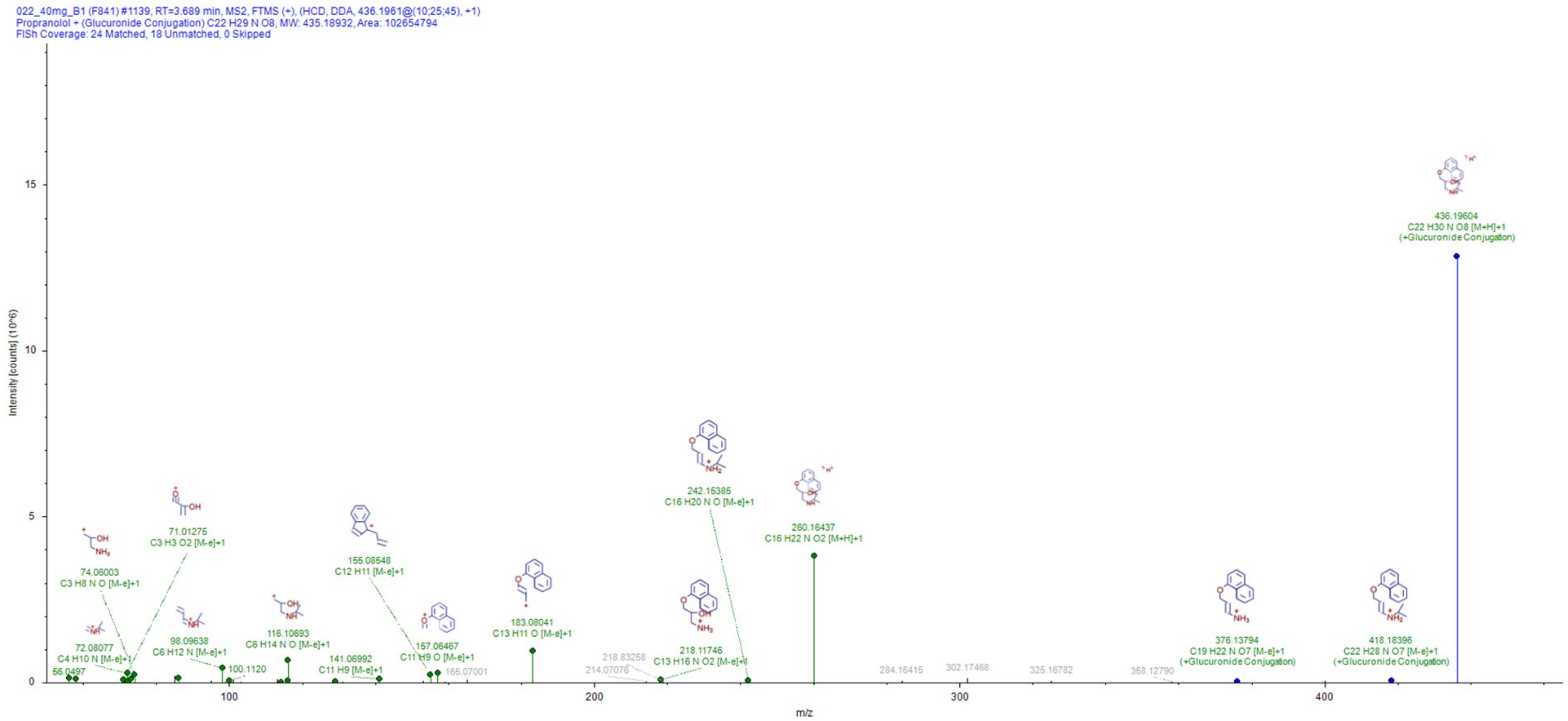

Figure S13. FISh scoring of feature 435.18914 (PPL-Glucu; HILIC) from Compound Discoverer.

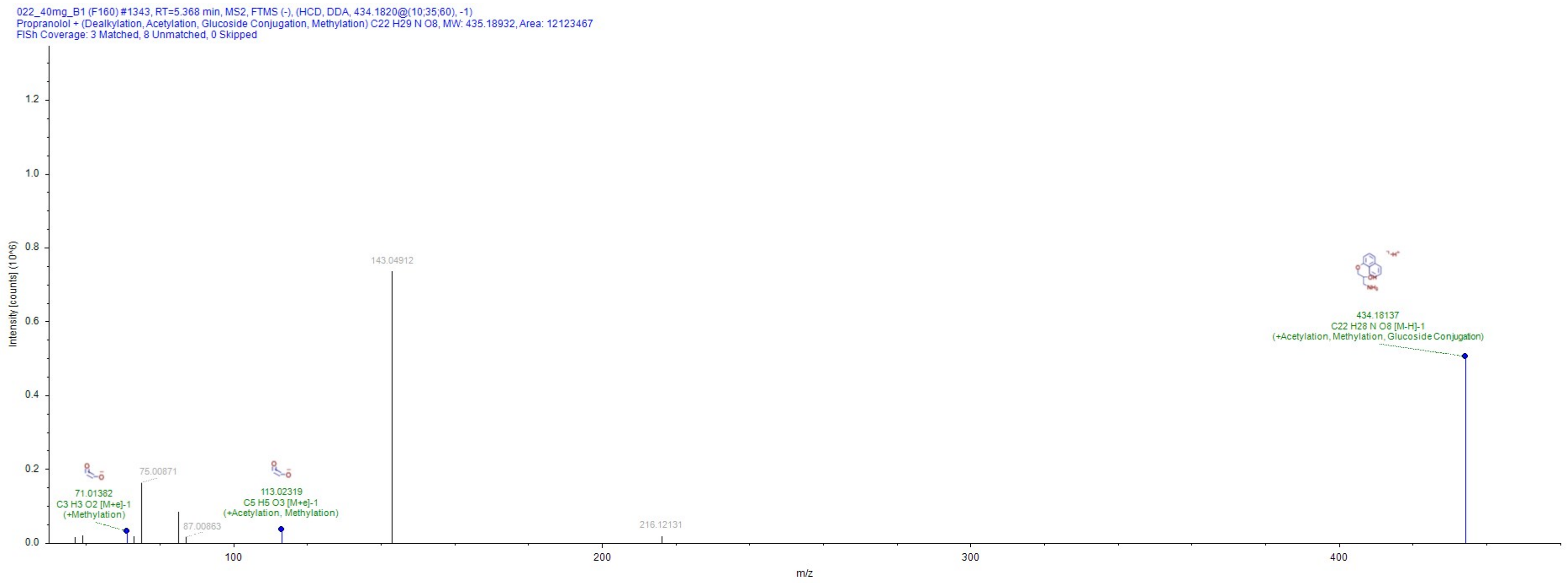

Figure S14. FISh scoring of feature 435.18914 (PPL-Glucu; C18) from Compound Discoverer.

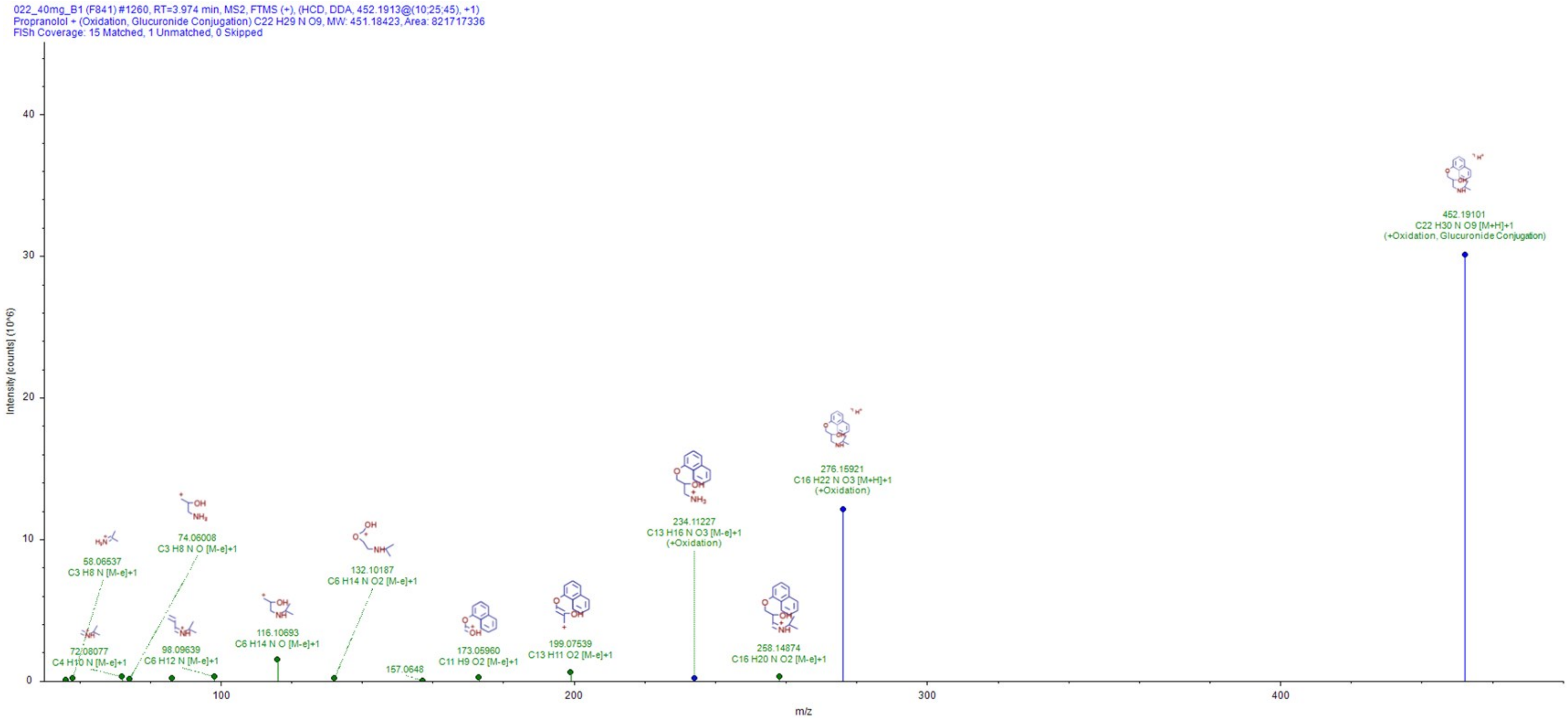

Figure S15. FISh scoring of feature 451.18423 (PPL-OH-Glucu; HILIC) from Compound Discoverer.

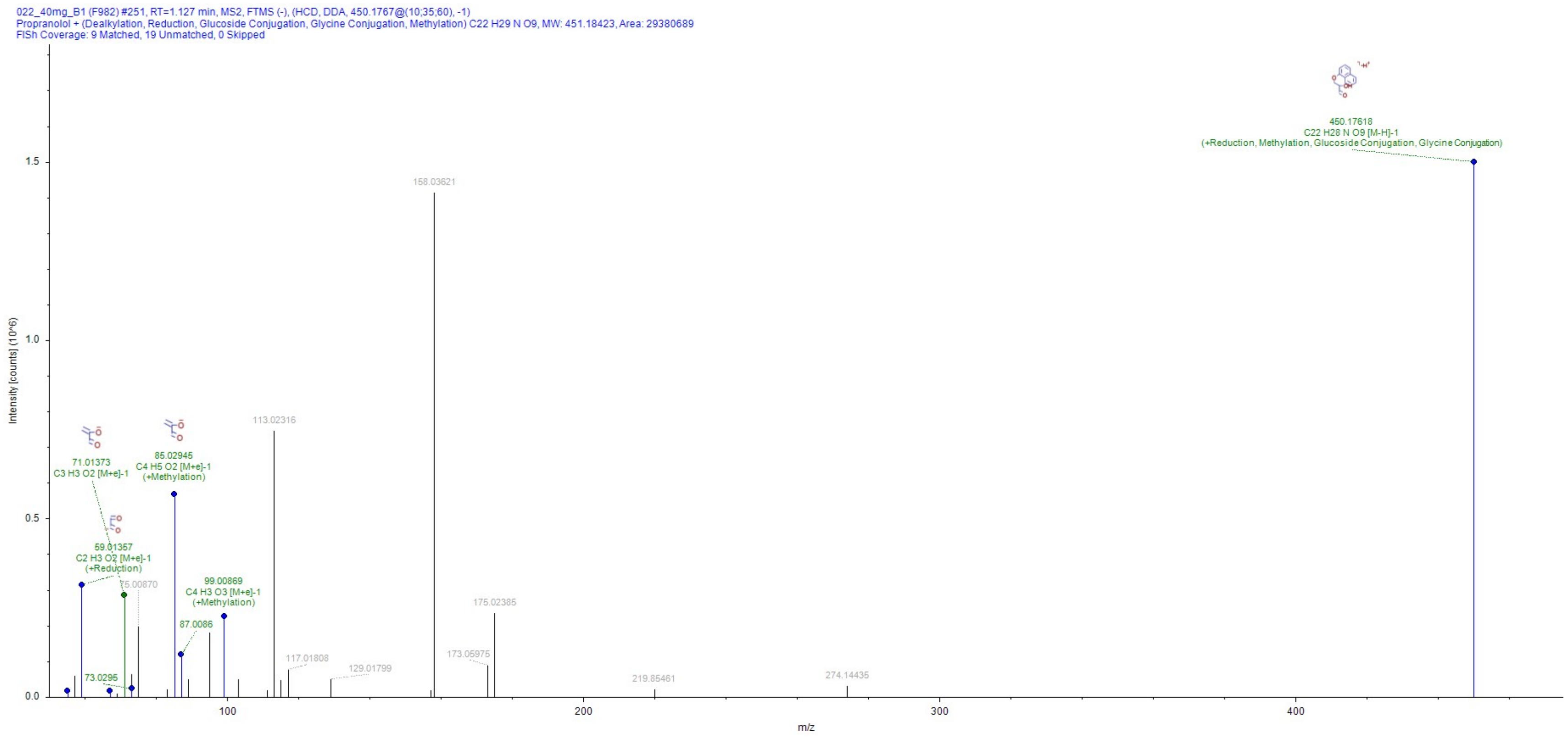

Figure S16. FISh scoring of feature 451.18407 (PPL-OH-Glucu; C18), peak RT 1.100 from Compound Discoverer.

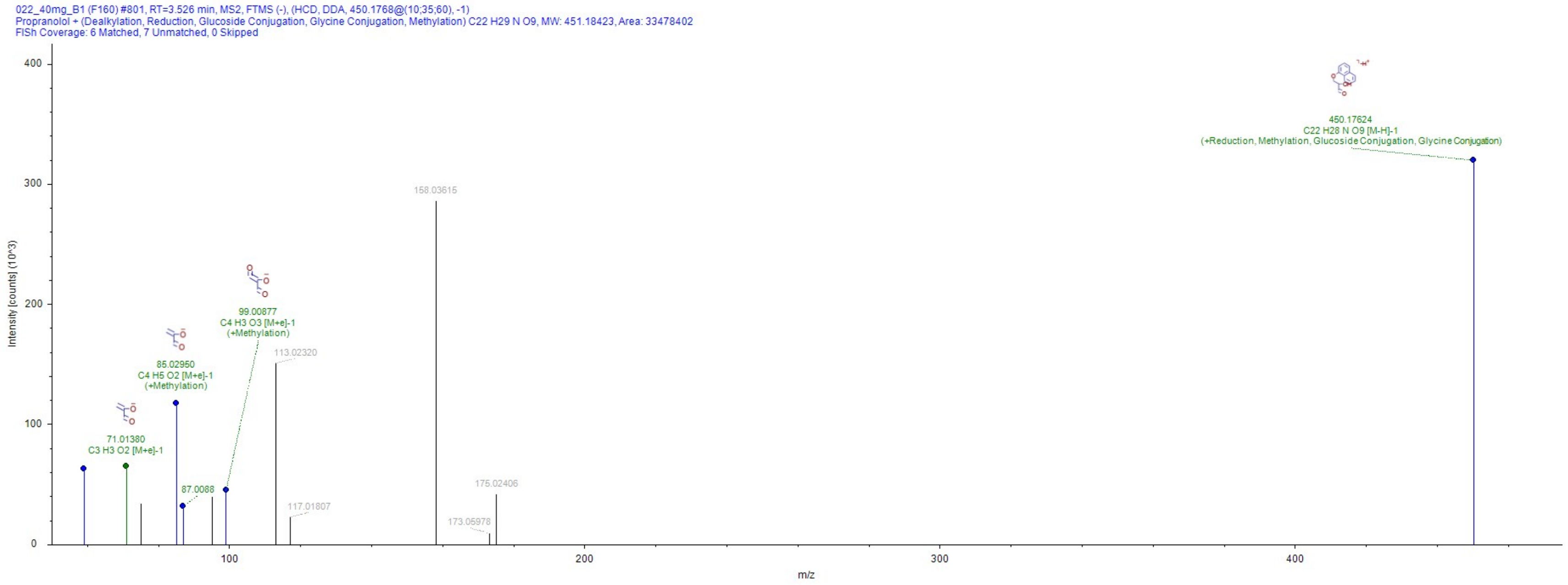

Figure S17. FISh scoring of feature 451.18407 (PPL-OH-Glucu; C18), peak RT 3.591 from Compound Discoverer.

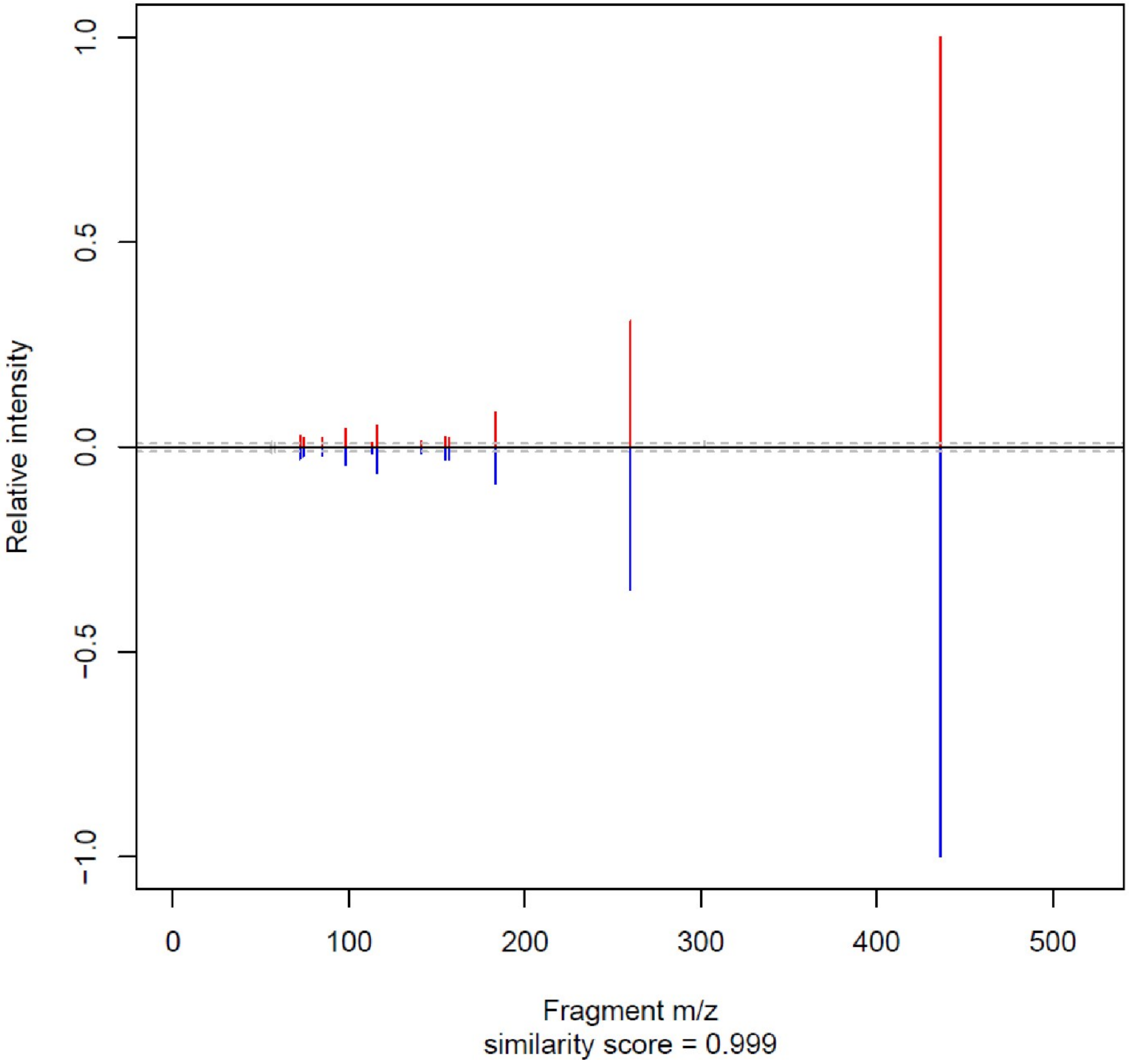

Figure S18. Similarity score from MSMSsim analysis of MS2 spectra of PPL-Glucu (HILIC) from a sample (upper half) and an authentic standard (lower half).

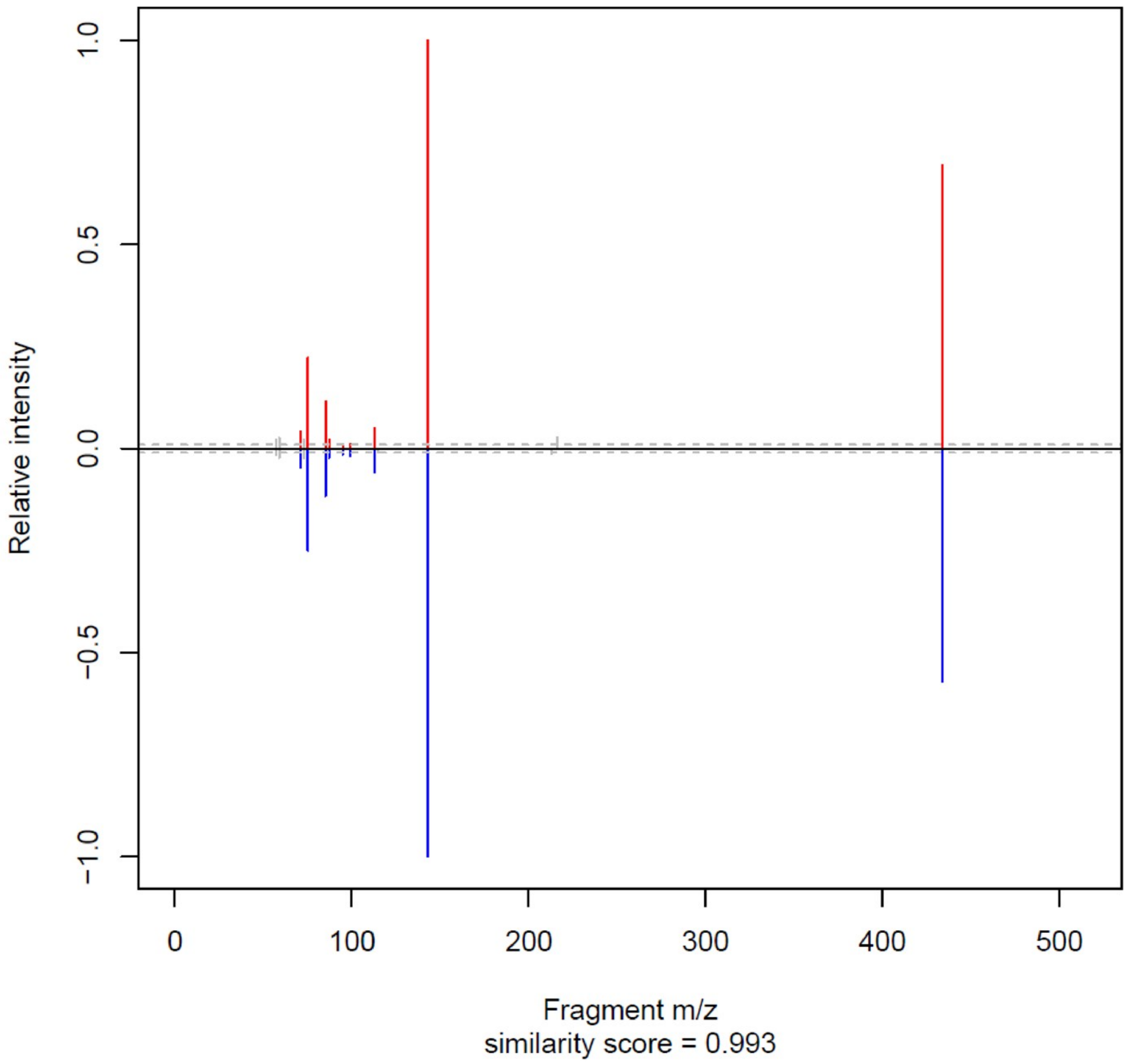

Figure S19. Similarity score from MSMSsim analysis of MS2 spectra of PPL-Glucu (C18) from a sample (upper half) and an authentic standard (lower half).

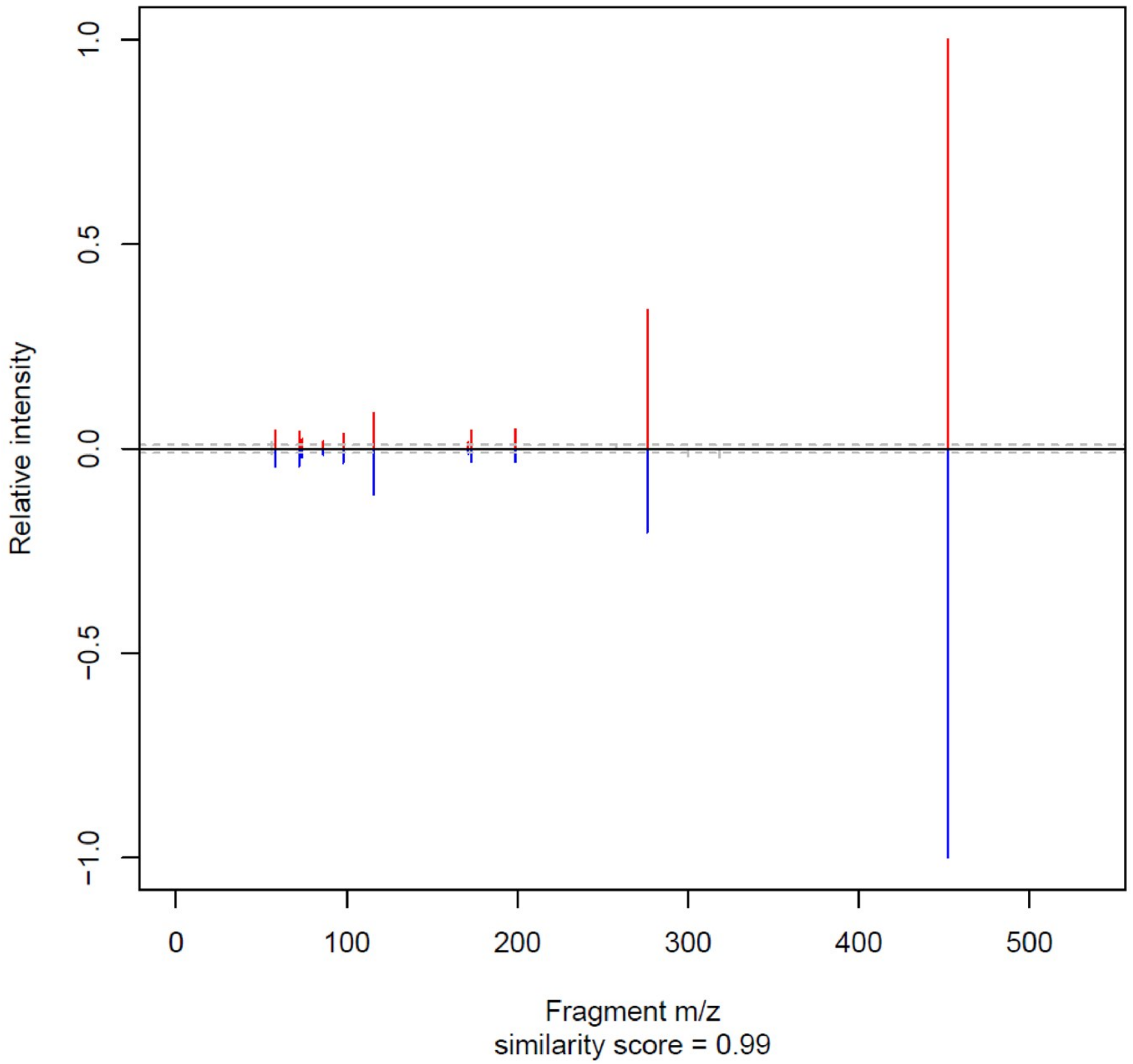

Figure S20. Similarity score from MSMSsim analysis of MS2 spectra of PPL-OH-Glucu (HILIC) from a sample (upper half) and an authentic standard (lower half).

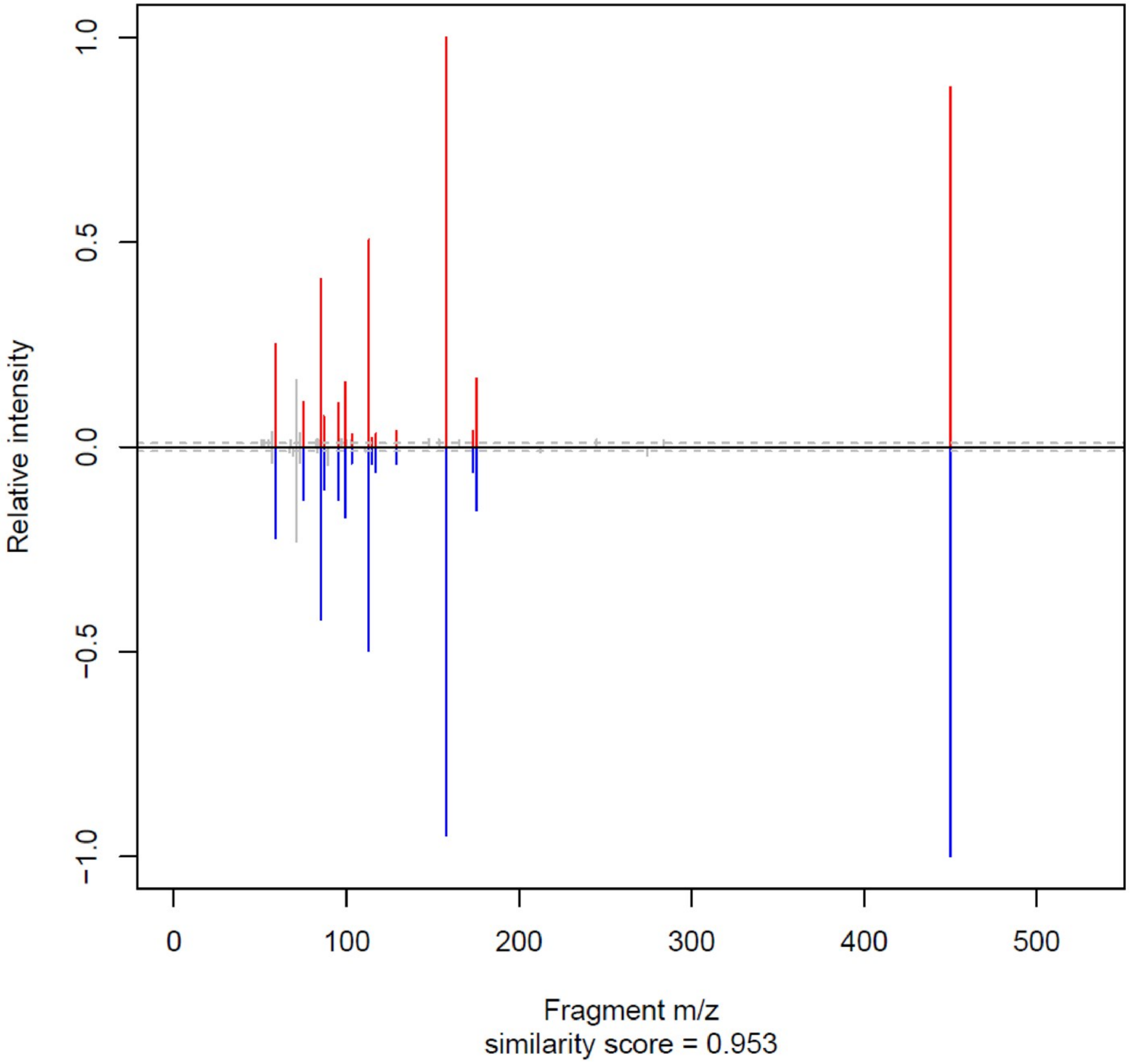

Figure S21. Similarity score from MSMSsim analysis of MS2 spectra of PPL-OH-Glucu (HILIC) from a sample (upper half) and an authentic standard (lower half).

Table S1. Sirius+CSI:FingerID (v4.5.1) settings for identification of features. All settings not mentioned were left at default.

**Parameter**	**Setting**
**SIRIUS**	
Instrument	Orbitrap
MS/MS isotope scorer	IGNORE
MS2 MassDev (ppm)	5
Candidates	10
Candidates per ion	1
Consider only formulas in DBs	All included DBs
Possible ionizations	[M+H]+, [M+K]+, [M+Na]+
**ZODIAC**	
All settings	Default
**CSI:FingerID**	
Search in DBs	All included DBs
Fallback adducts	[M+H]+, [M+K]+, [M+Na]+

Table S2. MSMSsim script settings for MS2 comparisons between authentic standards and samples which varied between features. All settings not mentioned in the table were the same for all features and were: ppm tolerance = 5, relative intensity cutoff = 0.01. All settings not mentioned were left at default.

Peak annotation	Ion mode	RT (min)	m/z tolerance	RT window (sec)
DIP-PPL	Pos(HILIC)	3.2	0.0015	20
PPL-4OH	Pos(HILIC)	3.07	0.001	10
PPL-5OH	Pos(HILIC)	3.18	0.0012	10
PPL-OH-SO4	Pos(HILIC)	3.2	0.00098	20
PPL-OH-SO4	Neg(C18)	4.1	0.0025	30
PPL-Glucu	Pos(HILIC)	3.5	0.00175	30
PPL-Glucu	Neg(C18)	3.6	0.0015	30
PPL-OH-Glucu	Pos(HILIC)	3.7	0.0015	30
PPL-OH-Glucu	Neg(C18)	5.4	0.0015	30

Text S1. Biotransformation product analysis workflow settings from Compound Discoverer

Search name: HILICPPLMetabNewSettings
Search description: Untargeted Metabolomics workflow: Retention time alignment, Component Detection, Grouping, Elemental Composition Prediction, Gap Filling, Hide chemical Background (using blanks), ID using mzCloud (needs MS/MS) and ChemSpider (using Formula); KEGG Pathway Mapping and Differential Analysis (ANOVA, adjusted p-values, fold change, CV, etc.)
Search date: 3/9/2020 4:29:52 PM
Created with Discoverer version: 3.1.0.305

```
[Input Files (0)]
    -->Select Spectra (36)

    [Select Spectra (36)]
        -->Align Retention Times (2)

        [Align Retention Times (2)]
            -->Detect Compounds (3)
            -->Find Expected Compounds (27)

        [Generate Expected Compounds (32)]
            -->Find Expected Compounds (27)

            [Detect Compounds (3)]
                -->Group Compounds (23)

            [Find Expected Compounds (27)]
                -->Group Expected Compounds (33)

                [Group Compounds (23)]
                    -->Fill Gaps (21)
                    -->Search mzCloud (37)
                    -->Assign Compound Annotations (40)
                    -->Search Mass Lists (38)
                    -->Search mzVault (39)
                    -->Mark Background Compounds (24)
                    -->Search ChemSpider (10)
                    -->Predict Compositions (8)

                [Group Expected Compounds (33)]
                    -->FISh Scoring (34)
                    -->Mark Background Compounds (35)
                    -->Search mzCloud (37)
                    -->Search Mass Lists (38)
                    -->Search mzVault (39)
```

[Fill Gaps (21)]

[Search mzCloud (37)]

[Assign Compound Annotations (40)]

[Search Mass Lists (38)]

[Search mzVault (39)]

[Mark Background Compounds (24)]

[Search ChemSpider (10)]

[Predict Compositions (8)]

[FISh Scoring (34)]

[Mark Background Compounds (35)]

[Differential Analysis (12)]

Processing node 0: Input Files

Input Data:
- File Name(s) (Hidden):
    D:\Anton\ZFE\180531 - HILIC\PlateB\001_QC_BA2.raw
    D:\Anton\ZFE\180531 - HILIC\PlateB\002_QC_BA2.raw
    D:\Anton\ZFE\180531 - HILIC\PlateB\003_QC_BA2.raw
    D:\Anton\ZFE\180531 - HILIC\PlateB\004_40ug_D4.raw
    Etc. Etc.

Processing node 36: Select Spectra
----
1. General Settings:
- Precursor Selection: Use MS(n - 1) Precursor
- Use Isotope Pattern in Precursor Reevaluation: True
- Provide Profile Spectra: Automatic
- Store Chromatograms: False

2. Spectrum Properties Filter:
- Lower RT Limit: 0
- Upper RT Limit: 0
- First Scan: 0

- Last Scan: 0
- Ignore Specified Scans: (not specified)
- Lowest Charge State: 0
- Highest Charge State: 0
- Min. Precursor Mass: 0 Da
- Max. Precursor Mass: 5000 Da
- Total Intensity Threshold: 0
- Minimum Peak Count: 1

3. Scan Event Filters:
- Mass Analyzer: (not specified)
- MS Order: Any
- Activation Type: (not specified)
- Min. Collision Energy: 0
- Max. Collision Energy: 1000
- Scan Type: Any
- Polarity Mode: (not specified)

4. Peak Filters:
- S/N Threshold (FT-only): 1.5

5. Replacements for Unrecognized Properties:
- Unrecognized Charge Replacements: 1
- Unrecognized Mass Analyzer Replacements: ITMS
- Unrecognized MS Order Replacements: MS2
- Unrecognized Activation Type Replacements: CID
- Unrecognized Polarity Replacements: +
- Unrecognized MS Resolution@200 Replacements: 60000
- Unrecognized MSn Resolution@200 Replacements: 30000

Processing node 2: Align Retention Times
----
1. General Settings:
- Alignment Model: Adaptive curve
- Alignment Fallback: Use Linear Model
- Maximum Shift [min]: 1.5
- Shift Reference File: True
- Mass Tolerance: 4 ppm
- Remove Outlier: True

Processing node 3: Detect Compounds
----
1. General Settings:
- Mass Tolerance [ppm]: 4 ppm

- Intensity Tolerance [%]: 30
- S/N Threshold: 3
- Min. Peak Intensity: 120000
- Ions: [M+2H]+2; [M+ACN+H]+1; [M+H]+1; [M+H-H2O]+1
- Base Ions: [M+H]+1; [M-H]-1
- Min. Element Counts: C
- Max. Element Counts: C90 [13]C H190 Br3 Cl4 D5 K2 N10 [15]N Na2 O15 P2 S5

2. Peak Detection:
- Filter Peaks: True
- Max. Peak Width [min]: 1
- Remove Singlets: True
- Min. # Scans per Peak: 10
- Min. # Isotopes: 1

Processing node 23: Group Compounds
----
1. Compound Consolidation:
- Mass Tolerance: 4 ppm
- RT Tolerance [min]: 1

2. Fragment Data Selection:
- Preferred Ions: [M+H]+1; [M-H]-1

Processing node 21: Fill Gaps
----
1. General Settings:
- Mass Tolerance: 4 ppm
- S/N Threshold: 8
- Use Real Peak Detection: True

Processing node 37: Search mzCloud
----
1. General Settings:
- Compound Classes: All
- Precursor Mass Tolerance: 10 ppm
- FT Fragment Mass Tolerance: 10 ppm
- IT Fragment Mass Tolerance: 0.4 Da
- Library: Autoprocessed; Reference
- Post Processing: Recalibrated
- Max. # Results: 10
- Annotate Matching Fragments: False

2. DDA Search:
- Identity Search: HighChem HighRes
- Match Activation Type: True
- Match Activation Energy: Match with Tolerance
- Activation Energy Tolerance: 20
- Apply Intensity Threshold: True
- Similarity Search: None
- Match Factor Threshold: 60

3. DIA Search:
- Use DIA Scans for Search: False
- Max. Isolation Width [Da]: 500
- Match Activation Type: False
- Match Activation Energy: Any
- Activation Energy Tolerance: 100
- Apply Intensity Threshold: False
- Match Factor Threshold: 20

Processing node 40: Assign Compound Annotations
----
1. General Settings:
- Mass Tolerance: 5 ppm

2. Data Sources:
- Data Source #1: mzCloud Search
- Data Source #2: Predicted Compositions
- Data Source #3: MassList Search
- Data Source #4: ChemSpider Search
- Data Source #5: (not specified)
- Data Source #6: (not specified)
- Data Source #7: (not specified)

3. Scoring Rules:
- Use mzLogic: True
- Use Spectral Distance: True
- SFit Threshold: 20
- SFit Range: 20

Processing node 38: Search Mass Lists
----
1. Search Settings:
- Mass Lists: Endogenous Metabolites database 4400 compounds.massList
- Mass Tolerance: 5 ppm
- Use Retention Time: True

- RT Tolerance [min]: 2

Processing node 39: Search mzVault
----
1. Search Settings:
- mzVault Library: Custom mzVault Library.db
- Max. # Results: 10
- Match Factor Threshold: 50
- Search Algorithm: HighChem HighRes
- Match Analyzer Type: True
- IT Fragment Mass Tolerance: 0.4 Da
- FT Fragment Mass Tolerance: 10 ppm
- Use Retention Time: False
- Precursor Mass Tolerance: 10 ppm
- Apply Intensity Threshold: True
- Match Ionization Method: True
- Ion Activation Energy Tolerance: 20
- Match Ion Activation Energy: Match with Tolerance
- Match Ion Activation Type: True
- Compound Classes: All
- Remove Precursor Ion: True
- RT Tolerance [min]: 2

Processing node 24: Mark Background Compounds
----
1. General Settings:
- Max. Sample/Blank: 5
- Max. Blank/Sample: 0
- Hide Background: False

Processing node 10: Search ChemSpider
----
1. Search Settings:
- Database(s): KEGG; LipidMAPS
- Search Mode: By Formula or Mass
- Mass Tolerance: 5 ppm
- Max. # of results per compound: 100
- Max. # of Predicted Compositions to be searched per Compound: 3
- Result Order (for Max. # of results per compound): Order By Reference Count (DESC)

2. Predicted Composition Annotation:
- Check All Predicted Compositions: True

Processing node 8: Predict Compositions
----
1. Prediction Settings:
- Mass Tolerance: 4 ppm
- Min. Element Counts: C H
- Max. Element Counts: C90 H190 Br3 Cl4 K2 N10 Na2 O15 P2 S5
- Min. RDBE: -1
- Max. RDBE: 40
- Min. H/C: 0.1
- Max. H/C: 3
- Max. # Candidates: 10
- Max. # Internal Candidates: 200

2. Pattern Matching:
- Intensity Tolerance [%]: 30
- Intensity Threshold [%]: 0.1
- S/N Threshold: 3
- Min. Spectral Fit [%]: 10
- Min. Pattern Cov. [%]: 90
- Use Dynamic Recalibration: True

3. Fragments Matching:
- Use Fragments Matching: True
- Mass Tolerance: 5 ppm
- S/N Threshold: 3

Processing node 27: Find Expected Compounds
----
1. General Settings:
- Mass Tolerance: 5 ppm
- Intensity Tolerance [%]: 40
- Intensity Threshold [%]: 0.1
- SN Threshold: 3
- Min. # Isotopes: 2
- Min. Peak Intensity: 100000
- Average Peak Width [min]: 0

Processing node 33: Group Expected Compounds
----
1. Compound Consolidation:
- RT Tolerance [min]: 0.5

2. Fragment Data Selection:

- Preferred Ions: [M-2H]-2; [M-H]-1; [M-H-H2O]-1

Processing node 34: FISh Scoring
----
1. General Settings:
- Annotate Full Tree: True
- Match Transformations: True
- S/N Threshold: 3
- High Acc. Mass Tolerance: 2.5 mmu
- Low Acc. Mass Tolerance: 0.5 Da

2. Fragment Prediction Settings:
- Use General Rules: True
- Use Libraries: True
- Max. Depth: 5
- Aromatic Cleavage: True
- Min. Fragment m/z: 50

Processing node 35: Mark Background Compounds
----
1. General Settings:
- Max. Sample/Blank: 5
- Max. Blank/Sample: 0
- Hide Background: True

Processing node 32: Generate Expected Compounds
----
1. Compound Selection:
- Compounds: propranolol (C16 H21 N O2)

2. Dealkylation:
- Apply Dealkylation: True
- Apply Dearylation: True
- Max. # Steps: 1
- Min. Mass [Da]: 120

3. Transformations:
- Phase I:
    Dehydration (H2 O ->)
    Desaturation (H2 ->)
    Hydration (-> H2 O)
    Nitro Reduction (O2 -> H2)
    Oxidation (-> O)

Oxidative Deamination to Alcohol (H2 N -> H O)
Oxidative Deamination to Ketone (H3 N -> O)
Reduction (-> H2)
Thiourea to Urea (S -> O)

- Phase II:
  Acetylation (H -> C2 H3 O)
  Arginine Conjugation (H O -> C6 H13 N4 O2)
  Cysteine Conjugation 1 (H -> C3 H6 N O2 S)
  Cysteine Conjugation 2 (-> C3 H7 N O2 S)
  Glucoside Conjugation (H -> C6 H11 O5)
  Glucuronide Conjugation (H -> C6 H9 O6)
  Glutamine Conjugation (H O -> C5 H9 N2 O3)
  Glycine Conjugation (H O -> C2 H4 N O2)
  GSH Conjugation 1 (-> C10 H15 N3 O6 S)
  GSH Conjugation 2 (-> C10 H17 N3 O6 S)
  Methylation (H -> C H3)
  Ornitine Conjugation (H O -> C5 H11 N2 O2)
  Palmitoyl Conjugation (H -> C16 H31 O)
  Stearyl Conjugation (H -> C18 H35 O)
  Sulfation (H -> H O3 S)
  Taurine Conjugation (H O -> C2 H6 N O3 S)
- Others: (not specified)
- Max. # Phase II: 3
- Max. # All Steps: 5

4. Ionization:
- Ions: [M+H]+1

Processing node 12: Differential Analysis
----
1. General Settings:
- Log10 Transform Values: True
